# Supplementary material for: A coherent phonon-induced hidden quadrupolar ordered state in Ca2RuO4
Source: Nat Commun. 2023 Dec 13;14:8258. doi: 10.1038/s41467-023-44021-4 (PMC10716257; doi:10.1038/s41467-023-44021-4)
Supplement: Supplementary file 1 — Supplementary Information [file 41467_2023_44021_MOESM1_ESM.pdf]

Supplementary Information to "A coherent phonon-induced hidden quadrupolar ordered state in Ca_2RuO_4"

Honglie Ning,[1,2,*] Omar Mehio,[1,2,*] Xinwei Li,[1,2,*] Michael Buchhold,[3]
Mathias Driesse,[2,†] Hengdi Zhao,[4] Gang Cao,[4] and David Hsieh[1,2,‡]
[1]*Institute for Quantum Information and Matter, California Institute of Technology, Pasadena, CA, USA*
[2]*Department of Physics, California Institute of Technology, Pasadena, CA, USA*
[3]*Institut für Theoretische Physik, Universität zu Köln, Cologne, Germany*
[4]*Department of Physics, University of Colorado, Boulder, Colorado 80309, USA*

Contents

Supplementary Note 1. Microscopic picture of the quadrupolar order in Ca_2RuO_4 2
Supplementary Note 2. Fitting of transient reflectivity traces 4
Supplementary Note 3. 2.5 THz hump in FFT spectra 5
Supplementary Note 4. Fluence dependence of phonon frequencies 6
Supplementary Note 5. Static and transient optical conductivity spectra 7
Supplementary Note 6. Temperature-dependent datasets 8
Supplementary Note 7. Estimate of laser heating 11
Supplementary Note 8. Details of the microscopic model for Ca_2RuO_4 12
Supplementary Note 9. Simulated equations of motion 16
Supplementary Note 10. Possible collective lattice distortion induced by QO switching 20
Supplementary Note 11. Probe-energy-dependent datasets 21
Supplementary Note 12. Density functional theory simulation results 23
Supplementary Note 13. Relationship between the A_g coherent phonons and octahedral eigenmodes Q's 24
Supplementary Note 14. Justification of the impulsive excitation of QOCP 26
Supplementary Note 15. Temperature, probe energy, and fluence dependence of the QOCP phase 27
Supplementary Note 16. Estimate of the light-induced atomic displacement 29
Supplementary Note 17. Robustness of the QO switch against nonlinear doublon-holon generation 30
References 31

Supplementary Note 1. Microscopic picture of the quadrupolar order in Ca_2RuO_4

Here we elaborate on the previously reported mechanism underlying quadrupolar ordering and related physics in Ca_2RuO_4. We start with constructing the low-energy electronic structure of Ca_2RuO_4 based on the relevant energy scales (Fig. S1). The dominant cubic field $10Dq \sim 3$ eV splits the higher-energy e_g manifold from the t_{2g} manifold. The effective orbital angular momentum of the sixfold degenerate t_{2g} manifold, which includes d_{xz}, d_{yz}, and d_{xy} orbitals, can be mapped directly to the $L = 1$ p-orbital, dubbed the T-P equivalence $\hat{\mathbf{L}}_{\mathbf{eff}}(\mathbf{t}_{2g}) = -\hat{\mathbf{L}}(\mathbf{p})$ [1].

Hund's coupling and spin-orbit coupling (SOC) then jointly determine the effective total angular momentum configuration of electrons in the t_{2g} manifold. Since the intra-atomic Hund's coupling $J_{\mathrm{H}} \sim 0.4$ eV is much smaller than $10Dq$, the four d-electrons will only occupy the t_{2g} manifold and favor a state with total spin $S = 1$. The moderate SOC $\lambda \sim 0.05 - 0.13$ eV endows the ground state with a total angular momentum $J = L \pm S$, with its constituents S and L in (anti)parallel fashion for the less (more) than half-filled t_{2g} manifold according to Hund's rules [2]. Since four electrons occupy the sixfold degenerate t_{2g} manifold, the ground state is a $J = L - S = 0$ singlet, with a $J = 1$ triplet and a $J = 2$ quintet at higher energies separated by λ and 3λ from the ground state, respectively. This ground state is ostensibly nonmagnetic, but a Van-Vleck excitonic magnetism involving a virtual transition from $J = 0$ to $J = 1$ can be facilitated when the SOC gap λ between the two levels is comparable to the inter-atomic magnetic exchange interaction κ [3]. This excitonic magnetic order hosts unique Goldstone and Higgs magnon modes, which have been experimentally confirmed by inelastic neutron scattering measurements [4].

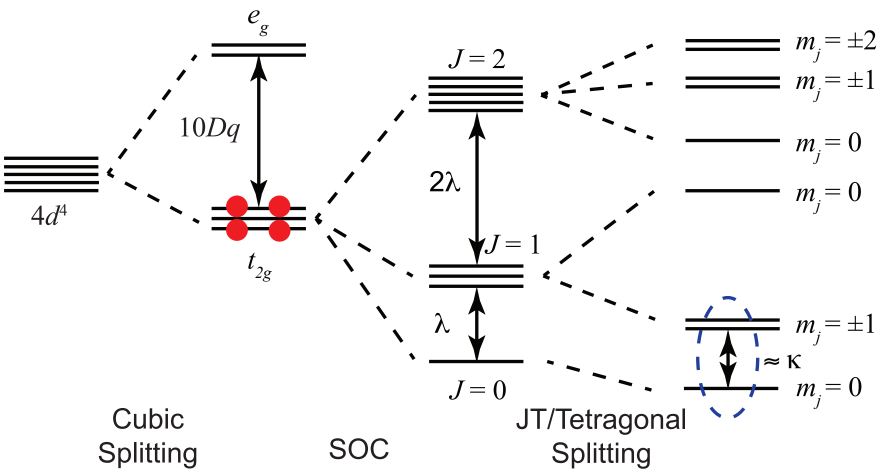

Fig. S1. Schematic of the energy levels for $4d^4$ multi-orbital Mott insulator Ca_2RuO_4. Cubic field $10Dq$ splits the d-shell into e_g and t_{2g} manifolds. The four electrons within the t_{2g} manifold, which are represented by the four red circles, favor a ground state with a total angular momentum $J = 0$ and two excited states with $J = 1$ and $J = 2$ in the presence of SOC. The excited states will be further split by tetragonal/JT distortion. When the gap between the $J = 0$ and $J = 1, m_j = \pm 1$ manifolds is comparable to magnetic exchange interaction κ, an excitonic condensation occurs, as shown by the blue dashed circle.

However, previous X-ray/optical/electron spectroscopy studies commonly find that $\kappa \sim 0.01 - 0.05$ eV, which is slightly smaller than λ [4–9]. This energy mismatch can be reconciled by a moderate Jahn Teller (JT)-type tetragonal splitting in the t_{2g}^4 manifold. JT effect, arising from the coupling between orbital and lattice degrees of freedom, drives a structural phase transition concomitant with the orbital ordering and thus removes the orbital degeneracy. Again, we consider the case with four electrons in the t_{2g} manifold. When $\lambda = 0$, two electrons with anti-parallel spins will occupy the d_{xy} orbital and the other two electrons with parallel spins will occupy $d_{xz/yz}$ orbitals. Consequently, the octahedron will exhibit a compression along the z-axis. In the presence of a moderate SOC, as is the case for Ca_2RuO_4, JT effect still exhibits a finite effect [10]. The $J = 1$ manifold will split into a doublet with $m_j = \pm 1$ and a singlet with $m_j = 0$, and the $J = 2$ manifold will split into a singlet with $m_j = 0$ and two doublets with $m_j = \pm 1$ and $m_j = \pm 2$, respectively (Fig. S1). The tetragonal splitting thus reduces the SOC gap between the $J = 0$ and $J = 1, m_j = \pm 1$ manifolds. When the gap is comparable to κ, an excitonic condensation occurs through virtual excitation from the $J = 0$ to the $J = 1, m_j = \pm 1$ state. Simultaneously, the lattice is compressed along the z-axis, inheriting from the $J = 1, m_j = \pm 1$ manifold. However, in the quasi-2D $J = 0$ system, owing to XY-type fluctuations, the magnetic ordering is suppressed to a temperature T_{N} — below the quadruopolar ordering temperature T_{QO} (see the last section of Ref. [11]). This gives rise to an intermediate time-reversal symmetric state with pseudospin quadrupolar order,

accompanied by the tetragonal compression [12, 13]. This JT-driven intermediate state, dubbed a spin-nematic, was previously interpreted as a d_{xy} ferro-orbital-ordered phase [14]. Also note that the magnetic transition does not necessarily accompany any rotation symmetry breaking. In addition, note that SOC cannot be too strong, or else the JT distortion will be fully suppressed and the excitonic magnetism will be quenched. See Note 8 and Ref.[10] for a more detailed discussion.

The specific case for Ca_2RuO_4 is a bit more subtle. The octahedron exhibits tetragonality instead of cubicity even above T_{QO}, so only a subtle lattice change accompanies the quadrupolar ordering without explicit symmetry breaking. Although challenging to detect using X-ray diffraction [15], this subtle change manifests as a quadrupolar-order-coupled phonon phase flip as captured by a recent coherent phonon spectroscopy study [16]. Our experiments further employ this coupled phonon to drive and detect a light-induced quadrupolar order transition.

Supplementary Note 2. Fitting of transient reflectivity traces

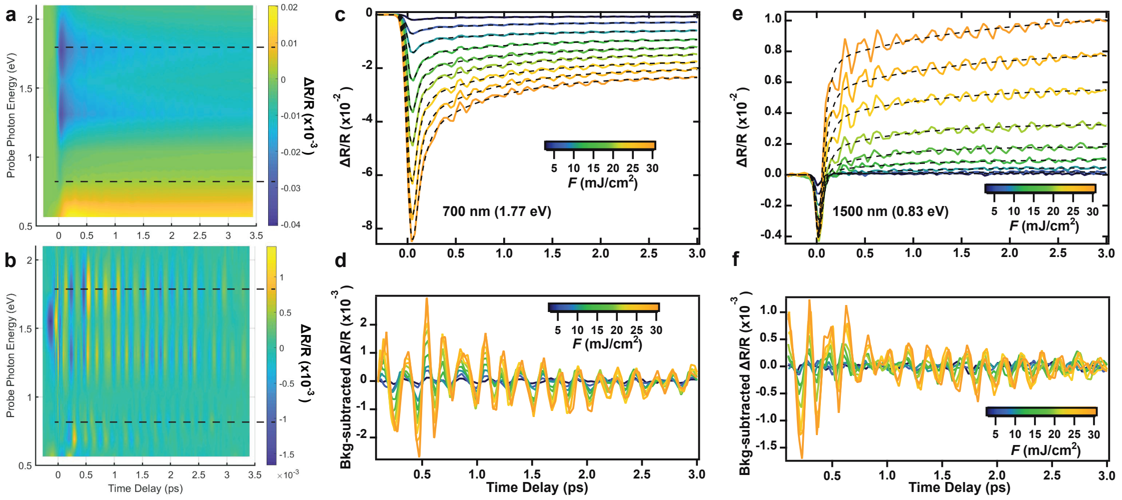

Fig. S2. **a,b,** Probe-energy-resolved transient reflectivity spectra before and after subtraction of the exponential background acquired with a pump fluence of 15 mJ/cm^2 at 80 K. The dashed lines denote the probe energies that are used to measure the curves as shown in **c-f**. **c,d,** Pump fluence-dependent reflectivity transients before and after subtraction of the double-exponential decaying background with a probe photon energy of 1.77 eV acquired at 80 K. **e,f,** Same as **c,d** but with a probe of 0.83 eV. The dashed lines represent fitting of background.

Here we summarize how we fit and subtract the background terms in reflectivity transients. A full probe-energy-dependent transient reflectivity spectrum at a pump fluence $F = 15$ mJ/cm^2 is shown in Fig. S2a. Two characteristic sets of fluence-dependent reflectivity transients probed at 1.77 eV and 0.83 eV are shown in Figs. S2c and e. There is a clear beat pattern that signifies the presence of multiple coherent phonons as well as a background that arises from charge excitation and relaxation. The background can be fit by a double-exponential decay or an exponential increase followed by an exponential decay plus a constant. We can in principle fit these time traces with a combination of damped oscillatory terms to characterize the phonons and exponential terms representing the background, which is convolved with a Guassian to account for the instrumental response function:

$$\frac{\Delta R}{R} = g(\sigma, t) * [\sum_{i=1,2} A_i \exp\left(-\frac{t}{t_i}\right) + C + \sum_j B_j \exp\left(-\frac{t}{\tau_j}\right) \cos\left(2\pi\nu_j t + \phi_j\right)] \tag{S1}$$

where $g(\sigma, t) = \exp\left(-\frac{4\ln(2)t^2}{\sigma^2}\right)$ is a Gaussian kernel that captures the instrumental temporal resolution $\sigma = 0.1$ ps, A_i denotes the amplitude of the decay background, t_i represents the corresponding decay (rise) time, C is a constant background that we observed within our time window, which may represent a slow heat diffusion process, and B_j, τ_j, ν_j, and ϕ_j are the amplitude, lifetime, frequency, and phase of the j-th phonon, respectively.

Since the coexistence of multiple phonons is hard to disentangle and thus fit in the time domain, we first ignore the damped oscillations and fit only the exponential background with $g(\sigma, t) * [\sum_{i=1,2} A_i \exp\left(-\frac{t}{t_i}\right) + C]$ (dashed lines in Figs. S2c and e). We then subtract this background term from the time domain traces and isolate the oscillatory components (Figs. S2d and f). This background-subtracted response is then converted into the frequency domain by a fast Fourier transform (FFT). Since distinct phonons emerge as discrete peaks in the frequency domain, by performing a multi-Lorentzian fit to the FFT spectra we can obtain their B_j, τ_j, and ν_j as a function of pump fluence, temperature, and probe-energy.

Note that the signs of A_i and C are dependent on the probe energy, and their amplitudes increase with pump fluence. In general, we see the following dependence. A more comprehensive discussion can be found elsewhere [17]:

Probe energy (eV)	A_1	A_2	C
1.2 - 2.2	< 0	< 0	< 0
0.8 - 1.1	< 0	< 0	> 0
0.55 - 0.7	< 0	> 0	> 0

Supplementary Note 3. 2.5 THz hump in FFT spectra

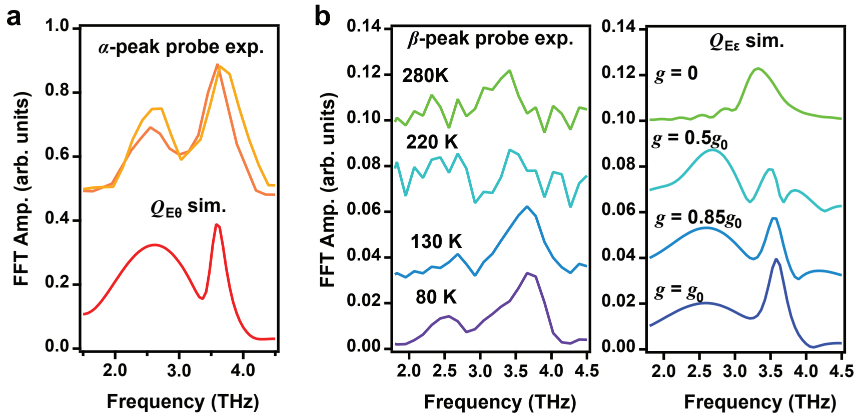

Fig. S3. **a,** FFT spectra of QOCP acquired from simulation and experiment with probe energies at 0.69 eV (yellow) and 0.92 eV (orange), which are located at the rising edge of the α-peak at 80 K. **b,** FFT spectra of QOCP acquired from simulation and experiment with a probe energy of 1.55 eV, which is located at the rising edge of the β-peak, at various temperatures. All the experimental data were taken at a pump fluence of 15 mJ/cm^2. The simulated FFT spectra are calculated by changing g in a form of $\sqrt{1 - T/T_{\mathrm{QO}}}$. The normalized values of g at each temperature are denoted. Both experimental and simulation curves are scaled and offset vertically for better comparison.

In both our experimental results and numerical simulations (Notes 6 & 9), there exists a hump at the low-frequency shoulder of the QOCP peak. According to previous Raman spectroscopy results [18], all the A_g modes have been identified and are located above 3.7 THz. Since our reflectivity measurements are mostly sensitive to A_g phonons according to symmetry, we can rule out the possibility that this hump is a phonon. Also, this hump exists even above T_{N} but disappears at T_{QO}, indicating that it is not a magnon but should be related to QO.

We postulate that the hump feature may arise from the anharmonicity of the potential energy surface (PES) as the state is transiently switched from one minimum to the other. When the PES is perfectly harmonic, the state will oscillate at a single frequency determined by the constant curvature. However, as SOC reduces the curvature of the barrier partitioning different local minima, the system will exhibit an oscillation with a lower energy component emerging when it is transiently switched between the minima. We would thus expect the emergence of low frequency humps in both $Q_{E\theta}$ and $Q_{E\epsilon}$ at $T < T_{\mathrm{QO}}$ (note that for simplicity we drop the letter "E" in all the formulas in the main text, whereas in the supplementary information we keep "E" for clarity. See a more detailed discussion in Note 8). Measurements acquired with probe energies located at the rising edges of the $d_{xy} \rightarrow d_{xz/yz}$ (α-peak) and $d_{xz/yz} \rightarrow d_{xz/yz}$ (β-peak) transitions and at different temperatures across T_{QO} show good agreement with the simulations (Fig. S3), supporting this hypothesis.

Supplementary Note 4. Fluence dependence of phonon frequencies

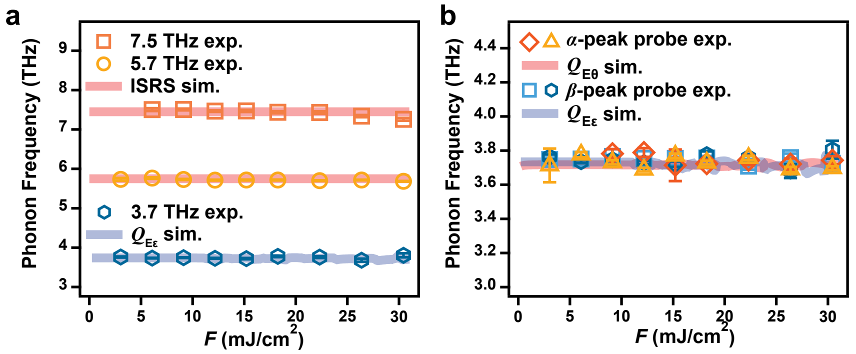

Fig. S4. Fluence dependence of QOCP and ISRS phonon frequencies obtained from experimental data and numerical simulations. The experimental data are acquired with 1.55 eV probe at 80 K. The simulation results are shifted vertically to match the experimental frequencies of different modes. The error bars are obtained from the standard deviation of the multi-Lorentzian fitting to the experimental FFT spectra.

While the amplitudes of both the QOCP and the QO-uncoupled phonons demonstrate a strong dependence on fluence, their frequencies show negligible change as the fluence is varied. A typical dataset is shown in Fig. S4a, which was captured at a probe energy of 1.55 eV. This behavior contrasts sharply with the equilibrium temperature dependence of the phonons, where changes larger than 0.5 THz can be resolved between 80 K and 300 K [16]. Importantly, there is no anomaly of QOCP frequency at $F_c = 15$ mJ/cm^2. This fluence independence is further corroborated by our numerical simulations of the QOCP and the QO-uncoupled phonons (see Note 9 for further details and the simulation results in Fig. S4a). Note, since the latter are generated by the impulsively stimulated Raman scattering (ISRS) mechanism, we refer to them as ISRS phonons.

In Fig. S4b, we find that the frequencies of the $Q_{E\theta}$ and $Q_{E\epsilon}$ components of the 3.7 THz QOCP (see Note 9 for further details) show a similar lack of fluence dependence with no drastic change at F_c. Note that a subtle softening of the 7.5 THz phonon with pump fluence may originate from carrier-excitation-induced lattice softening and phonon anharmonicity [19–21], which are ignored in our microscopic model.

Supplementary Note 5. Static and transient optical conductivity spectra

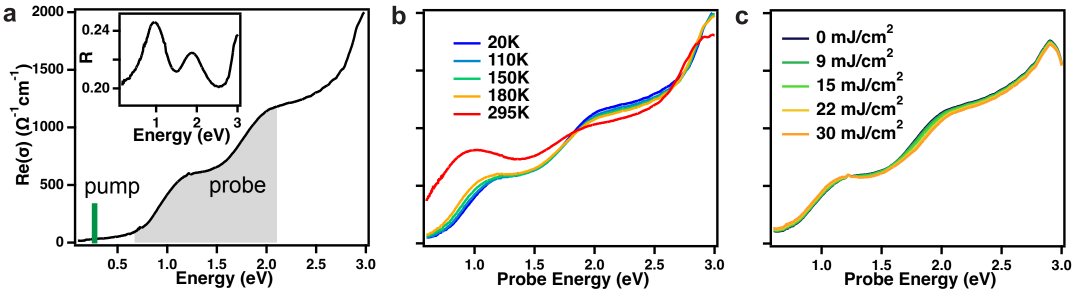

Fig. S5. **a,** Static optical conductivity and reflectivity spectra at 20 K. The 0.3 eV pump energy is marked by a vertical green line, while the probe energy range is shaded gray. **b,** Static optical conductivity as a function of temperature. **c,** Transient optical conductivity as a function of pump fluence measured at $t = 0$

.

We digitized the static optical conductivity of Ca_2RuO_4 from Ref.[16]. The equilibrium optical conductivity unambiguously shows that our pump energy is well below the low temperature Mott gap and far from any phonon resonances, which exist at energies lower than the 0.1 eV lower limit of the spectrum (Fig. S5a). The optical conductivity exhibits little change with temperature until the temperature reaches room temperature, where the first absorption peak as well as the bandgap region gains significant spectral weight, because the insulator-to-metal transition temperature is approached (Fig. S5b). We then obtained the transient optical conductivity spectra by Kramers-Kronig analysis of our transient reflectivity spectra at various probe energies and fluences [17]. The light-induced change of spectral weight at the highest fluence is at least one order of magnitude smaller than the corresponding change between the static optical conductivity at 80 K and 300 K, which is still 50 K lower than the insulator-to-metal transition temperature (Figs. S5b and c) [22]. This demonstrates that an insulator-to-metal transition is not transiently induced upon driving the material, and the PES of the system is only weakly perturbed.

Supplementary Note 6. Temperature-dependent datasets

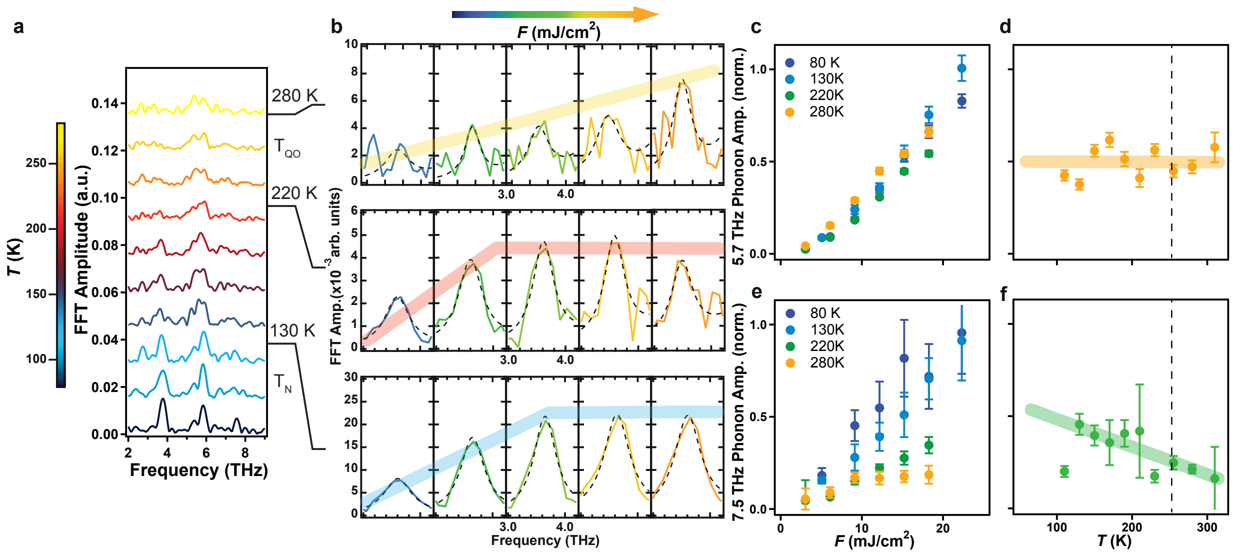

Fig. S6. **a,** Temperature dependent FFT spectra for 1.55 eV probe and 0.3 eV pump at 15 mJ/cm^2. Curves are offset vertically for clarity. **b,** Fluence dependence of FFT spectra at three characteristic temperatures as denoted in panel **a**. The dashed lines are Lorentzian fits and the thick colored lines are guides to the eye. **c,** Pump fluence dependence of the 5.7 THz phonon amplitude at select temperatures acquired with a 1.55 eV probe. All data are normalized to the maximal measured value. **d,** Temperature dependence of the 5.7 THz phonon amplitude measured at $F = 15$ mJ/cm^2 with a 1.55 eV probe. All data are normalized to the maximal measured value in **c**. The colored line is a guide to eye. **e** and **f** are the same as **c** and **d** but for the 7.5 THz mode. The dashed lines denote T_{QO}. The error bars are obtained from the standard deviation of the multi-Lorentzian fitting to the FFT spectra.

The temperature dependence of the FFT spectra measured at 1.55 eV and pumped with a constant fluence of 15 mJ/cm^2 at 0.3 eV is shown in Fig. S6a. All the phonon peaks exhibit a dramatic softening and broadening as temperature increases. The temperature dependence of the QOCP amplitude in Fig. 3b is obtained from these curves.

We conducted pump fluence dependence measurements at four characteristic temperatures: 80 K (below T_{N}), 130 K and 220 K (above T_{N} but below T_{QO}) and 280 K (above T_{QO}). The pump fluence dependence of the QOCP FFT spectra measured at 130 K, 220 K, and 280 K are displayed in Figs. S6b, while the data measured at 80 K is shown in Fig. 2d. The pump fluence dependence of the 3.7 THz mode amplitude in Fig. 3a is obtained from fits to these curves.

As a comparison, we present the fluence dependence of the QO-uncoupled 5.7 THz and 7.5 THz modes at 80, 130, 220, and 280 K as well as the temperature dependence of these modes measured at $F = 15$ mJ/cm^2 with a 1.55 eV probe in Figs. S6c-f, similar to the presentation for the 3.7 THz QOCP in Fig. 3. As expected, the quasi-linear pump fluence dependence persists at all the sampled temperatures for both modes, consistent with their QO-uncoupled nature. Moreover, the pump fluence dependence of the 5.7 THz mode measured at various temperatures almost follows a single line (Fig. S6c), supporting the temperature independence of the amplitude of the 5.7 THz mode (Fig. S6d). In contrast, the slope of the pump fluence dependence of the 7.5 THz mode, which reflects the photo-susceptibility at various temperatures, decreases with increasing temperature (Fig. S6e). This is also reflected in the temperature dependence of the 7.5 THz mode, where its amplitude decreases with increasing temperature, without any observable anomaly at T_{QO} (Fig. S6f), unlike the behavior observed for the 3.7 THz QOCP. These results provide additional confirmation that the 5.7 THz and 7.5 THz modes are not QOCP.

The data obtained with 0.3 eV pump at $F = 15$ mJ/cm^2 and a probe resonant with the β-transition does not suggest a clear upturn of the phonon amplitude above T_{QO}, but an upturn of phonon amplitude above T_{QO} with temperature was observed in Ref.[16]. We first acknowledge the difference in experimental conditions between our work and Ref. [16]. The experiment in Ref.[16] used a pump with a photon energy (1.55 eV) above the bandgap and a fluence range within the linear response regime ($F < 1$ mJ/cm^2). In contrast, our experiment employed a pump with a photon energy (0.3 eV) below the gap, enabling the highest fluence up to $F > 25$ mJ/cm^2 without thermally damaging the sample. To this end, Ref.[16] mainly explores the ground state properties with a relatively weak perturbation, while our work investigates the possible switch of QO in the strongly-driven out-of-equilibrium

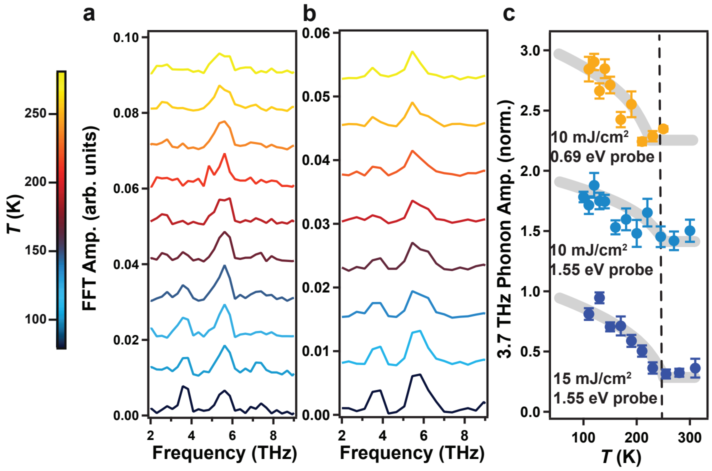

Fig. S7. **a,** Temperature dependent FFT spectra for 1.55 eV probe and 0.3 eV pump at 10 mJ/cm^2. Curves are offset vertically for clarity. **b,** Temperature dependent FFT spectra for 0.69 eV probe and 0.3 eV pump at 10 mJ/cm^2. Curves are offset vertically for clarity. **c,** Temperature dependence of the 3.7 THz phonon amplitude corrected for the pump-induced temperature rise obtained under different conditions as labelled in the legend. The thick colored lines are guides to the eye. The error bars are obtained from the standard deviation of the multi-Lorentzian fitting to the FFT spectra.

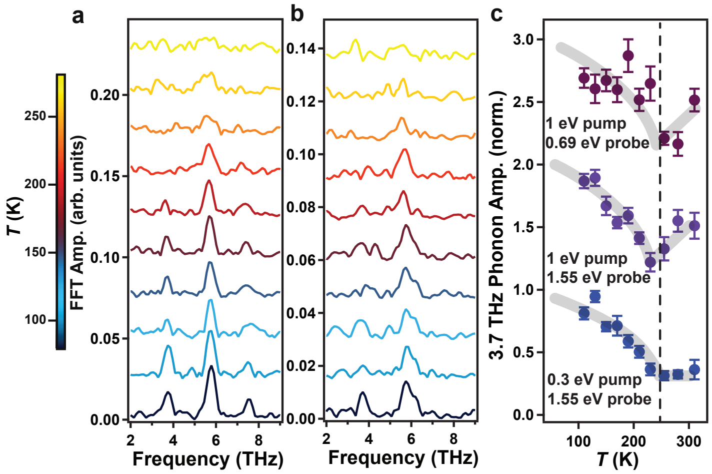

Fig. S8. **a,** Temperature dependent FFT spectra for 1.55 eV probe and 1 eV pump at 3 mJ/cm^2. Curves are offset vertically for clarity. **b,** Temperature dependent FFT spectra for 0.69 eV probe and 1 eV pump at 3 mJ/cm^2. Curves are offset vertically for clarity. **c,** Temperature dependence of the 3.7 THz phonon amplitude corrected for the pump-induced temperature rise obtained under different conditions as labelled in the legend. The thick colored lines are guides to the eye. The error bars are obtained from the standard deviation of the multi-Lorentzian fitting to the FFT spectra.

regime. Given the drastic difference in pump photon energy and fluence range, it expected that different results would be observed in the two studies.

To investigate whether the pump energy, pump fluence range, or probe energy is the primary cause of this distinction, we performed a series of temperature dependent measurements with different experimental conditions. We first repeat our temperature dependent measurements of the 3.7 THz mode at a lower pump fluence. We need to emphasize that a pump fluence smaller than F_c cannot realize the ultrafast switch to a hidden QO and the measured phonon amplitude cannot represent the maximal static JT distortion where the potential energy is minimized. Our temperature dependence measurements in the main text were conducted with a pump fluence slightly larger than F_c at 80 K, ensuring that the dynamical transition occurs at all measured temperatures. Therefore, in the new experiments we probe at 1.55 eV and pump with a moderately weak pump fluence of 10 mJ/cm^2, which is larger than F_c at $T > 150$ K and close to (albeit smaller than) F_c at $T < 150$ K (Fig. S7a). We find that the phonon amplitude shows a similar temperature dependence as that measured with a pump fluence of 15 mJ/cm^2 (Fig. S7c), suggesting that

the pump fluence may not play a dominant role in determining the temperature dependence of the QOCP amplitude and confirming that as long as the pump fluence is close to F_c, our results shown in the main text should qualitatively hold.

We then measured the temperature dependence of the QOCP with a probe energy at 0.69 eV, resonant with the rising edge of the α-peak, in addition to our current temperature dependence measured with a probe energy at 1.55 eV, resonant with the β-peak (Fig. S7b). The phonon amplitude in general shows a similar temperature dependence as that measured with a probe energy of 1.55 eV, albeit with a lower signal-to-noise ratio (Fig. S7c). This is expected in our microscopic model, because probe energy resonant with the α- and β-peaks is sensitive to Q_θ and Q_ϵ, respectively. Since both of them exhibit an upturn at T_{QO}, their temperature dependence should be qualitatively similar.

Finally, we measured the temperature dependence of the QOCP with an above-gap pump at a photon energy of 1 eV and $F = 3$ mJ/cm^2, which is nearly resonant with the α-peak, and probed with energies resonant with both α- and β-peaks. Interestingly, we observe a more evident upturn of phonon amplitude above T_{QO} with 1 eV pump compared to the 0.3 eV pump, resembling the results reported in Ref.[16] (Fig. S8). This may indicate that the pump photon energy plays a more important role in generating the different temperature dependence of QOCP above T_{QO}. This may be understood because subgap pumping and above-gap pumping have different excitation mechanisms. The phonon amplitude with a subgap pump (ISRS) is determined by $d\mathrm{Re}(\varepsilon)/\mathrm{dE}$, where ε is the complex dielectric constant and E is the photon energy, whereas the phonon amplitude with an above-gap pump (DECP) is determined by $\mathrm{Im}(\varepsilon)/\omega_{\mathrm{phonon}}$, which is related to the electronic absorption [23, 24]. These two tensors may have different temperature dependence. A clearer answer to this question needs more comprehensive pump energy and fluence dependent measurements, which are beyond the scope of the current work.

Supplementary Note 7. Estimate of laser heating

There are two types of heating that can occur in an impulsively driven system. The first is instantaneous heating induced by a single laser pulse, which can be estimated with the equation $\Delta T = \frac{(1-R)F}{C\rho\delta}$ [25], where ρ is the sample density, F is the fluence (we use the critical fluence $F_c = 15$ mJ/cm^2), R is the reflectivity of the sample at 0.3 eV [26], δ is the optical penetration depth of pump at 0.3 eV [26], and C is the heat capacity [27]. Using experimentally measured values for each of these parameters at 80 K, we determined that the largest effective temperature increase at 80 K is 5 K, which is negligable.

The second kind of heating, cumulative heating by the laser pulses, can cause a static temperature increase. Due to the quasi-2D structure of Ca_2RuO_4, the in-plane thermal conductivity is several times higher than the out-of-plane thermal conductivity [28]. Therefore, the temperature gradient induced by the laser will be mainly along the out-of-plane direction. The temperature increase can be estimated to be $\Delta T = \frac{Pd}{A\kappa}$, where P is the input power (1.5 mW at F_c), d is the thickness of the sample (~ 0.5 mm), A is the cross-section of the beam with a FWHM of 100 μm, and κ is the thermal conductivity along the c-axis (~ 1.8 W/(K·m)) [28]. Using these values, we obtain a steady temperature increase of around 30 K. Accordingly, we shift the data points in Fig. 3b by 30 K and find that the QOCP amplitude significantly increases at the reported T_{QO}.

Supplementary Note 8. Details of the microscopic model for Ca_2RuO_4

To quantitatively capture the physics of Ca_2RuO_4, we use the following microscopic single-ion model. Based on discussions in Note 1, we consider the t_{2g} manifold of electrons with SOC, JT/tetragonal distortion, and multi-orbital electronic interactions:

$$\hat{H} = \hat{H}_{\rm U} + \hat{H}_{\rm SOC} + \hat{H}_{\rm JT} + \hat{H}_{\rm L}, \tag{S2}$$

with

$$\begin{aligned} \hat{H}_{\rm JT} &= \sum_{\gamma=\theta,\epsilon} g Q_{E\gamma} \hat{\tau}_\gamma, \\ \hat{H}_{\rm L} &= \sum_{\gamma=\theta,\epsilon} \frac{1}{2} B Q_{E\gamma}^2, \\ \hat{H}_{\rm SOC} &= \lambda \hat{\mathbf{L}} \cdot \hat{\mathbf{S}}, \\ \hat{H}_{\rm U} &= (U - 3J_{\rm H}) \frac{\hat{N}(\hat{N}-1)}{2} + \frac{5}{2}\hat{N} - J_{\rm H}(2\hat{S}^2 + \hat{L}^2/2). \end{aligned} \tag{S3}$$

Here, λ is the SOC constant, g is the JT coupling constant, B is the elastic lattice energy that determines the QOCP frequency, $J_{\rm H}$ is the intra-atomic Hund's exchange, and U is the Hubbard intra-orbital Coulomb interaction. The Q's represent the two possible orthornormal eigenmodes of the octahedral complex with $E_{\rm g}$ symmetry. $\hat{L}$ and $\hat{S}$ are the total orbital and spin angular momentum operators, $\hat{N}$ is the total electron number operator, and $\hat{\tau}$ is the quadrupolar operator. We can then perform exact diagonalization with electron filling number $N = 4$. The lowest eigenvalue will capture the PES as a function of the two structural order parameters $Q_{E\theta}$ and $Q_{E\epsilon}$. We now elaborate on each term as follows.

Lattice-related terms

We first consider the lattice-related terms $\hat{H}_{\rm JT} + \hat{H}_{\rm L}$. Hereafter, we label the lattice irreproducible representation with capital letters to distinguish them from the electronic channel. According to the selection rule: $t_{2g} \otimes t_{2g} = A_{1g} \oplus E_g \oplus t_{2g}$, the t_{2g} electrons can interact with the fully symmetric A_{1g} mode, two tetragonal/orthorhombic E_g modes, and three trigonal t_{2g} modes. The spatial configurations of the six octahedral orthornormal eigenmodes are shown in Fig. S9. We adopt a nomenclature more widely used in JT community which denotes $\{d_{yz}, d_{xz}, d_{xy}, d_{3z^2-r^2}, d_{x^2-y^2}\}$ as $\{\xi, \eta, \zeta, \theta, \epsilon\}$ for simplicity [29].

The general JT Hamiltonian can be written as [30]:

$$\hat{H}_{\rm JT} = \sum_k \sum_{n\Lambda\lambda} \sum_{\Lambda_1\Lambda_2\cdots\Lambda_k} \frac{1}{k!} g_{n\Lambda}^{\Lambda_1\Lambda_2\cdots\Lambda_k} \times \{Q_{\Lambda_1} \otimes Q_{\Lambda_2} \otimes \cdots \otimes Q_{\Lambda_k}\}_{n\Lambda\lambda} \hat{\tau}_{\Lambda\lambda}. \tag{S4}$$

Here $\Lambda(\Lambda_i)$ is E_g or t_{2g}, λ is its component, n distinguishes the repeated representation, $Q_{\Lambda\lambda}$ is the orthornormal coordinate, $\{Q_{\Lambda_1} \otimes Q_{\Lambda_2} \otimes \cdots \otimes Q_{\Lambda_k}\}_{\Lambda\lambda}$ is the symmetrized product of coordinates, g is the k-th order coupling parameter, and $\hat{\tau}_{\Lambda\lambda}$ are the matrices of Clebsch-Gordan coefficients, which can be considered as quadrupolar angular momentum operators.

Only considering linear coupling terms, we can simplify the JT interaction as:

$$\hat{H}_{\rm JT} = \sum_{\gamma=\theta,\epsilon} g_E Q_{E\gamma} \hat{\tau}_{E\gamma} + \sum_{\gamma=\xi,\eta,\zeta} g_T Q_{T\gamma} \hat{\tau}_{T\gamma}. \tag{S5}$$

We also include the elastic lattice term:

$$\hat{H}_{\rm L} = \sum_{\gamma=\theta,\epsilon} \frac{1}{2} B_E Q_{E\gamma}^2 + \sum_{\gamma=\xi,\eta,\zeta} \frac{1}{2} B_T Q_{T\gamma}^2. \tag{S6}$$

Note that the T_{2g} and E_g eigenmodes in general have different microscopic parameters B and g.

The quadrupolar tensors $\hat{\tau}$ can be written out as a linear superposition of quadratures of orbital angular momentum operators depending on the specific symmetry of the coupled eigenmodes: $\hat{\tau}_{\mu\nu} = \frac{1}{2}(\hat{L}_\mu \hat{L}_\nu + \hat{L}_\nu \hat{L}_\mu) - \frac{L(L+1)}{3}\delta_{\mu\nu}$. Specifically, we have $\hat{\tau}_{E\theta} = -\frac{1}{2}(2\hat{L}_z^2 - \hat{L}_x^2 - \hat{L}_y^2)$, $\hat{\tau}_{E\epsilon} = -\frac{\sqrt{3}}{2}(\hat{L}_x^2 - \hat{L}_y^2)$, $\hat{\tau}_{T\xi} = -\frac{1}{\sqrt{2}}(\hat{L}_y\hat{L}_z + \hat{L}_z\hat{L}_y)$, $\hat{\tau}_{T\eta} = -\frac{1}{\sqrt{2}}(\hat{L}_x\hat{L}_z + \hat{L}_z\hat{L}_x)$, $\hat{\tau}_{T\zeta} = -\frac{1}{\sqrt{2}}(\hat{L}_x\hat{L}_y + \hat{L}_y\hat{L}_x)$.

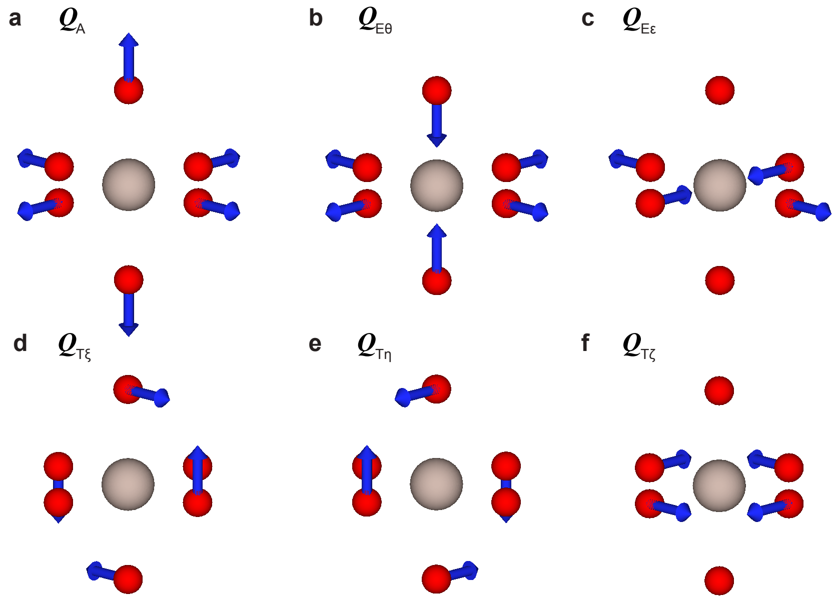

Fig. S9. Eigenmodes of the octahedron that can couple to t_{2g} electrons. **a**. Q_A, **b**. $Q_{E\theta}$, **c**. $Q_{E\epsilon}$, **d**. $Q_{T\xi}$, **e**. $Q_{T\eta}$, and **f**. $Q_{T\zeta}$. The red balls represent O atoms at the apices of the octahedra and the gray balls represent Ru atoms.

In Ca_2RuO_4, the t_{2g} distortion can be omitted because the coupling constant g_T is typically at least several times smaller than g_E in transition metal oxides without static trigonal distortions [12, 13, 30, 31]. Since the octahedra in Ca_2RuO_4 are tetragonal without any trigonal distortions within the temperature range of our study, the leading-order JT effect in Ca_2RuO_4 is indeed the E_g-distortions. Note that including t_{2g} distortions will only generate new energy minima in the PES. As the nonlinear fluence dependence of the phonon amplitude arises from the transient switching between different energy minima, including t_{2g} distortions will not qualitatively change the main conclusion of this paper. As shown in Fig. S11a, the value of both E_g and t_{2g} JT distortions shows a quantitatively similar dependence on SOC, indicating that as long as λ is not too large, the existence of multiple minima applies to both E_g and t_{2g} distorted cases, confirming the main conclusion of the paper. Note that for simplicity we drop the letter "E" in all the formulas in the main text, whereas in the supplementary information we keep "E" for clarity.

After neglecting t_{2g} distortions, we can plot the PES in the space formed by the two E_g eigenmodes $Q_{E\theta}$ and $Q_{E\epsilon}$. Without the JT interaction ($g_E = 0$), the PES is a paraboloid centered at $(Q_{E\theta}, Q_{E\epsilon}) = (0, 0)$ with zero lattice distortion and zero orbital polarization. In the presence of a finite JT coupling, the PES is composed of three identical paraboloids shifted away from the origin in three directions with angular separation of 120° (first panel of Fig. S10), indicating a $U(1)$-to-Z_3 symmetry breaking. The three equivalent minima at $(-\frac{g_E}{B_E}, 0), (\frac{g_E}{2B_E}, \frac{\sqrt{3}}{2B_E}), (\frac{g_E}{2B_E}, -\frac{\sqrt{3}}{2B_E})$ correspond to an identical tetragonal compression $u = \sqrt{Q_{E\theta}^2 + Q_{E\epsilon}^2} = \frac{g_E}{B_E}$ along z, y, and x-axis, respectively, and the electronic wavefunctions show the d_{xy}, d_{xz}, and d_{yz}-orbital character, respectively.

Adding a tetragonal splitting $\hat{H}_{\mathrm{TS}} = \Delta \hat{L}_z^2$ will elevate the d_{yz} and d_{xz} minima and leave the d_{xy} minimum as the global minimum, so that d_{xy} QO is uniquely reached, which is indeed the case for Ca_2RuO_4. We neglect this term in our dynamical simulation but show later in Note 9 that including such a term will not qualitatively change our main conclusion.

Spin-orbit coupling

Now, we include the SOC term $\hat{H}_{\mathrm{SOC}} = \lambda \hat{\mathbf{L}} \cdot \hat{\mathbf{S}}$. According to Ref.[10], whether JT competes or coexists with SOC depends on the electron filling number. For the single-electron-occupied case t_{2g}^1, we can analytically solve

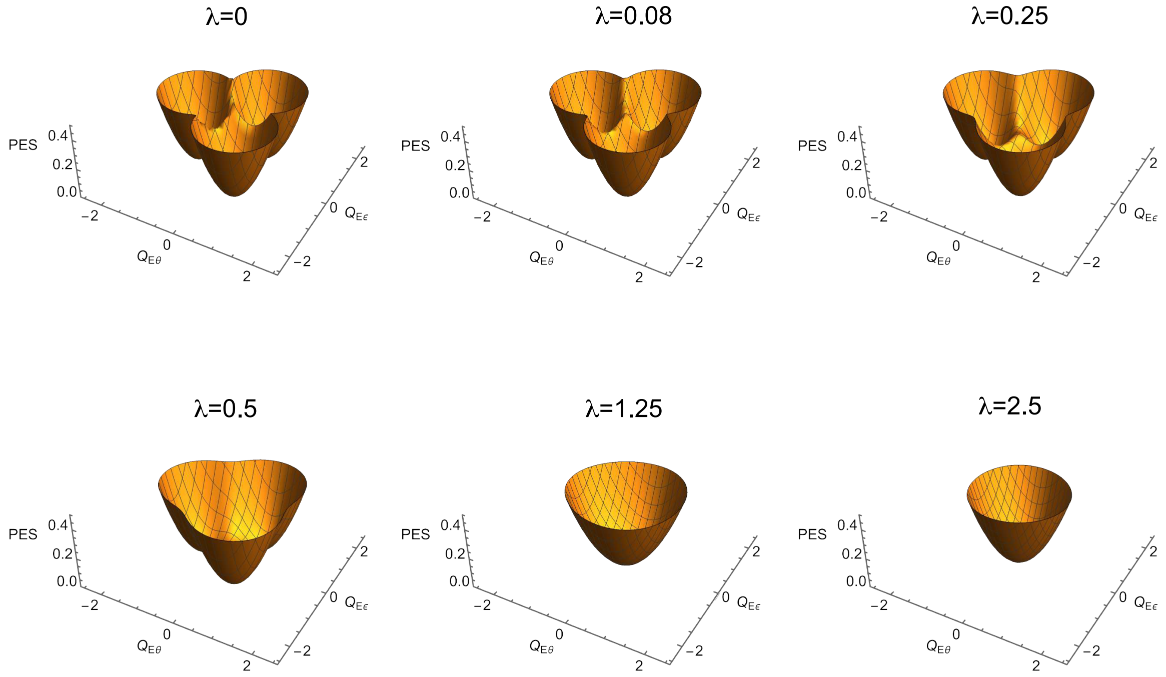

Fig. S10. Calculated PES as a function of λ for t_{2g}^4 case. The unit of the horizontal axes is g_E/B_E and the unit of the vertical axis is g_E^2/B_E. The unit of λ is g_E^2/B_E.

$\hat{H}_{\mathrm{JT,E}} + \hat{H}_{\mathrm{L,E}} + \hat{H}_{\mathrm{SOC}}$ and investigate the minimal JT distortion amplitude u^λ as a function of λ. u^λ shows a decrease with λ and asymptotically approaches half of $u^{\lambda=0}$ when $\lambda \to \infty$ (Fig. S11a) [10].

The evolution of the PES as a function of λ for the t_{2g}^4 case, obtained by numerically solving $\hat{H}_{\mathrm{SOC}} + \hat{H}_{\mathrm{JT,E}} + \hat{H}_{\mathrm{L,E}}$, is shown in Fig. S10. As λ increases, the three minima corresponding to compression along x, y, and z-axes become shallower and closer, similar to the t_{2g}^1 case. However, when λ is larger than a critical value λ_c, the PES recovers an isotropic parabolic geometry and the JT distortion vanishes, in contrast to the t_{2g}^1 case where the JT distortion is preserved even when $\lambda \to \infty$. Therefore, although SOC and the JT effect compete for both the d^1 and d^4 configurations, in the latter case SOC will fully suppress JT at $\lambda > \lambda_c$ (Fig. S11b).

Electronic interactions

We now consider the intra- and inter-orbital Coulomb interaction, spin exchange, and pair hopping within the t_{2g} manifold. This can be captured by a Kanamori multi-orbital electronic interaction term [2]:

$$\begin{aligned}\hat{H}_{\mathrm{U}} =& U\sum_i \hat{n}_{i\uparrow}\hat{n}_{i\downarrow} + U'\sum_{i\neq i'}\hat{n}_{i\uparrow}\hat{n}_{i'\downarrow} + (U'-J_{\mathrm{H}})\sum_{i<i',\sigma}\hat{n}_{i\sigma}\hat{n}_{i'\sigma} \\ &- J_{\mathrm{H}}\sum_{i\neq i'}\hat{c}^\dagger_{i\uparrow}\hat{c}_{i\downarrow}\hat{c}^\dagger_{i'\downarrow}\hat{c}_{i'\uparrow} + J_{\mathrm{H}}\sum_{i\neq i'}\hat{c}^\dagger_{i\uparrow}\hat{c}^\dagger_{i\downarrow}\hat{c}_{i'\downarrow}\hat{c}_{i'\uparrow}.\end{aligned} \tag{S7}$$

With $U' = U - 2J_{\mathrm{H}}$ and rotational invariance, the Kanamori Hamiltonian takes a simplified form as shown before:

$$\hat{H}_{\mathrm{U}} = (U - 3J_{\mathrm{H}})\frac{\hat{N}(\hat{N}-1)}{2} + \frac{5}{2}\hat{N} - J_{\mathrm{H}}(2\hat{S}^2 + \hat{L}^2/2). \tag{S8}$$

Note that in this form Hund's first two rules (maximal S and then maximal L) are explicitly fulfilled. Since we are dealing with the isolated ion case with a fixed number of electrons, we can safely set $U = 0$ [10].

We note that when λ is several times smaller than λ_c, the JT distortion in the d^4 case simulated by the comprehensive microscopic model is analogous to that of the d^1 case where $\hat{H}_{\mathrm{U}}$ does not contribute (Fig. S11b). The PES of the

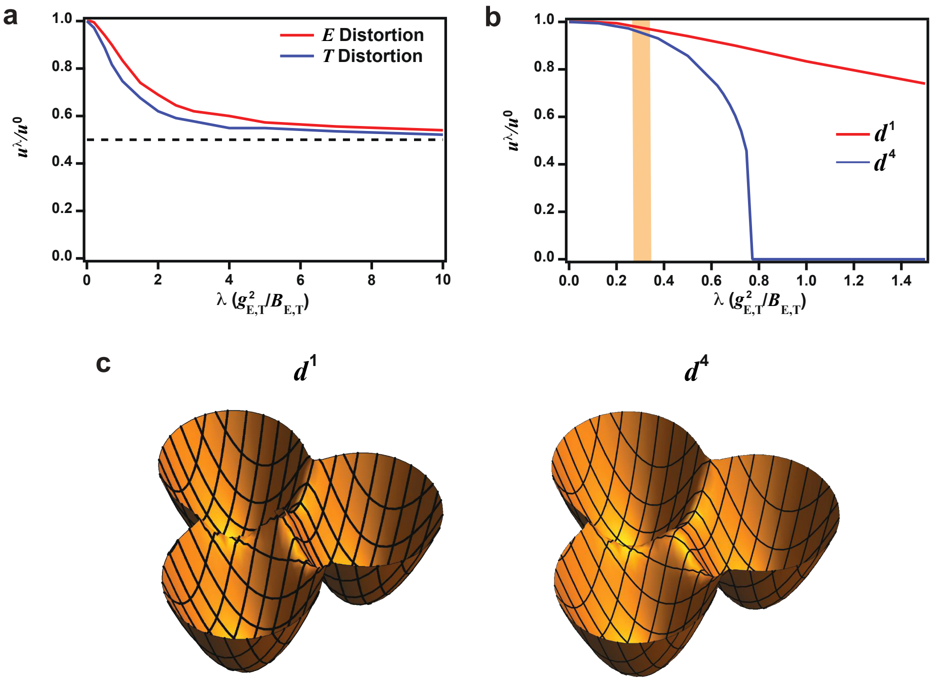

Fig. S11. **a,** JT distortion amplitude of E_g and t_{2g} distortions as a function of SOC in the d^1 electron configuration. **b,** E_g distortion amplitude as a function of SOC for d^1 and d^4 configurations. The orange bar denotes the value of λ for Ca_2RuO_4. **c,** PES of d^1 and d^4 cases. All the parameters are the same except for the electron filling number N.

d^1 and d^4 cases are also quantitatively similar (Fig. S11c). Therefore, completely excluding $\hat{H}_U$ and treating the problem as a single electron case can provide quantitatively similar results.

Supplementary Note 9. Simulated equations of motion

Simulation assumptions and parameter assignment

We denote the PES of the microscopic model described in Section S8 as $V(Q_{E\theta}, Q_{E\epsilon})$. We assume an impulsive excitation (see Supplementary Note 14 for more evidence). Since the pump photon energy is one order of magnitude larger than the energy of any of the observed phonons, we average out the sinusoidal oscillatory part of the pump but retain its Gaussian envelop with a time duration σ of 0.1 ps and an amplitude proportional to the pump fluence $F(t) = AF\exp\left[-\frac{4\ln(2)t^2}{\sigma^2}\right]$. Here, A is a scaling factor determined by the electron-phonon coupling and the real part of the dielectric constant at the pump energy [23]. Since $Q_{E\epsilon}$ and $Q_{E\theta}$ are decomposed from the same phonon, we assume they have equivalent A. We also include a phonon damping term with decay constant γ in the equations of motion of the $Q_{E\theta}$ and $Q_{E\epsilon}$ modes:

$$\frac{d^2Q_{E\theta/\epsilon}(t)}{dt^2} + 2\gamma\frac{dQ_{E\theta/\epsilon}(t)}{dt} + \frac{dV(Q_{E\theta}(t), Q_{E\epsilon}(t))}{dQ_{E\theta/\epsilon}(t)} = F(t) \tag{S9}$$

Since the phonon frequency is solely determined by B_E, we set $B_E = \omega_0 \sim (2\pi) \times 4$ THz. Based on previous X-ray/optical/electron spectroscopy results [4–8], we set $g_E = 2B_E$, $\lambda = 0.3\frac{g_E^2}{2B_E} = 0.3E_{JT,E}$. Therefore, the form of $V(Q_{E\theta}, Q_{E\epsilon})$ is pinned down. The damping constant γ is 0.4 THz based on our phonon spectroscopy measurement. Therefore, the effective pump fluence AF is the only tunable parameter. For the initial conditions, we set the system to stay in the d_{xy}-dominated minimum with zero initial velocity: $Q_{E\theta}(0) = Q_{E\theta}|_{eq} = -u_E^{\lambda}$, $\frac{dQ_{E\theta}(t)}{dt}|_{t=0} = 0$, $Q_{E\epsilon}(0) = Q_{E\epsilon}|_{eq} = 0$, and $\frac{dQ_{E\epsilon}(t)}{dt}|_{t=0} = 0$.

Simulation results

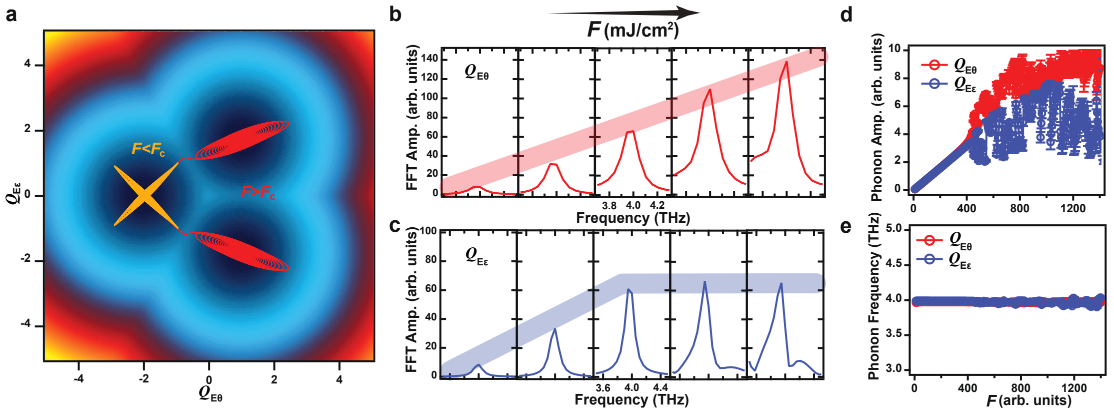

Fig. S12. **a,** Temporal trajectory of the QO parameter in ($Q_{E\theta}$,$Q_{E\epsilon}$) plane upon pumping with $F > F_c$ (red) and $F < F_c$ (yellow) atop a false-color equilibrium PES map. **b,** Fluence dependence of the FFT spectra of the QOCP projected onto the $Q_{E\theta}$ and **c** $Q_{E\epsilon}$ axes. **d,** Fluence dependence of the amplitude and **e,** frequency of the QOCP projected onto the the $Q_{E\theta}$ and $Q_{E\epsilon}$ axes. The error bars are obtained from the standard deviation of the multi-Lorentzian fitting to the FFT spectra acquired from the simulation.

The temporal evolution of the QO in the ($Q_{E\theta}$,$Q_{E\epsilon}$) plane upon pumping with two characteristic fluences ($F > F_c$ and $F < F_c$) is shown in Fig. S12. For $F > F_c$, the final state corresponds to a state with a different QO and compression along a different axis, as evidenced in Fig. S12a. The FFT of projections onto the Q_θ and Q_ϵ coordinates were then used to determine the coherent oscillation amplitudes (Figs. S12b and c). A fine sampling of fluence shows that $Q_{E\theta}$ exhibits a slight deviation from linear fluence scaling once $F > F_c$ but $Q_{E\epsilon}$ deviates much more drastically (Fig. S12d). Also note that although the QOCP amplitude shows an anomaly, its frequency is almost independent of F, since B_E, which determines the frequency of QOCP, is not F-dependent in our model (Fig. S12e).

There is an aperiodic oscillation of the QOCP amplitude as a function of fluence when $F > F_c$. This nonmonotonic behavior arises from switching between different minima as F increases. F_c thus corresponds to the fluence where the

first reversal occurs $F_{c,i=1}$. When F is slightly higher than F_{c1}, the system settles into the x/y-compressed minimum. When $F = F_{c2}$, the system will relax back to the original minimum. With even higher F, the system can switch between different minima transiently before it settles into one minimum.

In order to understand why switching between minima generates an aperiodic oscillation as a function of fluence, let us consider two fluences $F_1 = F_{c1} - \delta$ and $F_2 = F_{c1} + \delta$, where δ is a small positive value. As $F_2 > F_1$, the system will roll over the barrier and move to the new minimum. Since the system now travels a longer distance on the PES, damping will decrease the amplitude of the QOCP for the F_2 case more than the F_1 case. As F further increases, the larger energy pumped into the system will compensate the damping loss and the phonon amplitude will slowly increase until it reaches the boundary between the two minima again at $F = F_{c2}$. Then, the new reversal occurs and the phonon amplitude will drop again. Therefore, the QOCP amplitude will show a sudden change at each $F_{c,i}$, appearing as an oscillatory behavior as a function of F.

ISRS simulation

Conventional Raman active phonon modes can be coherently launched via the impulsive stimulated Raman scattering (ISRS) or displacive excitation of coherent phonons (DECP) mechanisms. The relationship between these two mechanisms has been extensively discussed elsewhere [23, 32–34]. In our subgap pump case, the ISRS mechanism dominates, allowing us to simulate the dynamics of the QO-uncoupled coherent phonons using the following formula [24]:

$$\partial_t{}^2 X = -\omega_{ph}{}^2 X - 2\gamma_{ph}\partial_t X + F(t), \tag{S10}$$

where $F(t) = F\exp\left[-\frac{4\ln(2)t^2}{\sigma^2}\right]$ with F being the normalized fluence. We can conduct an FFT analysis of the temporal evolution of the ISRS phonon and obtain its amplitude and frequency dependence as a function of pump fluence. As shown in Fig. 1c, the amplitude of the ISRS-launched phonon scales linearly with the pump fluence, while its frequency is a constant (Fig. S4a).

Including tetragonal splitting

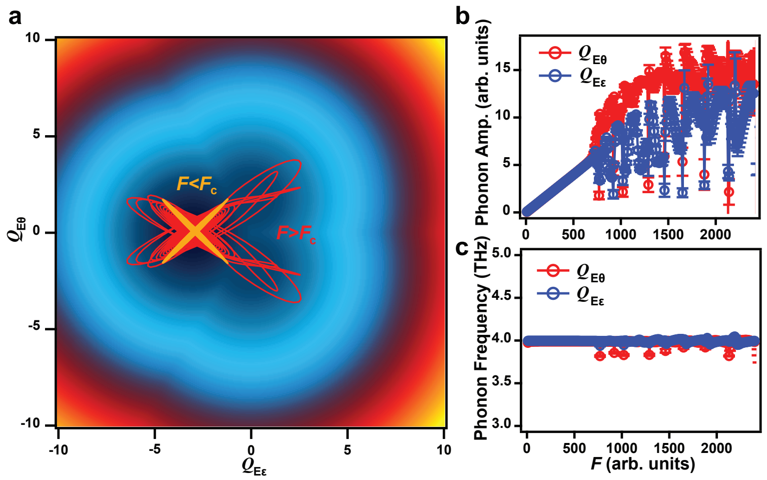

Fig. S13. **a,** Temporal trajectory in ($Q_{E\theta}$,$Q_{E\epsilon}$) plane upon a pump with $F > F_c$ (red) and $F < F_c$ (yellow) atop a false-color equilibrium PES map in the presence of tetragonal splitting. **b,** Fluence dependence of the amplitude and **c,** frequency of the QOCP projected onto the $Q_{E\theta}$ and $Q_{E\epsilon}$ axes. The error bars are obtained from the standard deviation of the multi-Lorentzian fitting to the FFT spectra acquired from the simulation.

We include the tetragonal splitting by adding a linear term $k_E Q_{E\theta}$ to tilt the PES so that the ground state of compressed z-axis is uniquely favored. The specific value of the slope is chosen such that the energy difference between the z-compressed global minimum and the x/y-compressed local minima is equal to the experimental tetragonal

splitting value obtained by X-ray scattering at temperatures higher than $T_{\rm QO}$ [5, 6]. The other parameters are kept unchanged. Accordingly, the PES will be tilted along the $Q_{E\theta}$ axis and the minima will be shifted. With this assumption, our system is now described by a pseudo-JT effect due to the energetic non-degeneracy in the parent phase [29]. However, this will not qualitatively affect the physics as explained below.

We show the dynamics of the system at two characteristic fluences above and below F_c. We find that, independent of F, the state will always settle into the original global minimum after about 3 ps (Fig. S13a), even though the switch between different QOs is transiently realized. Also note that F_c in the presence of tetragonal splitting is larger than that without tetragonal splitting because the d_{xz} and d_{yz} minima are elevated and thus harder to reach. However, despite the discrepancy, the fluence dependences of the QOCP amplitude and frequency are qualitatively identical to the case with tetragonal splitting ignored (Figs. S13b and c).

Temperature dependent simulation

We can simulate the temperature dependence of both the QOCP amplitude and F_c by assuming that g, which determines the spacing and depth of different minima in the PES, has an order-parameter-like temperature dependence with a mean-field critical exponent: $(1 - T/T_{\rm QO})^{\frac{1}{2}}$. The static JT distortion (Q_0) is suppressed as temperature increases and disappears at $T_{\rm QO}$. Our simulation confirms that Q_0 exhibits a primary order-parameter-like onset at $T_{\rm QO}$, validating our assumption on g (Fig. 3b). Similarly, our simulation shows that F_c exhibits an order-parameter-like onset at $T_{\rm QO}$ (Fig. 3c), in agreement with the experimental data. These results also justify the use of F_c as a direct measure of QO.

Robustness of the results

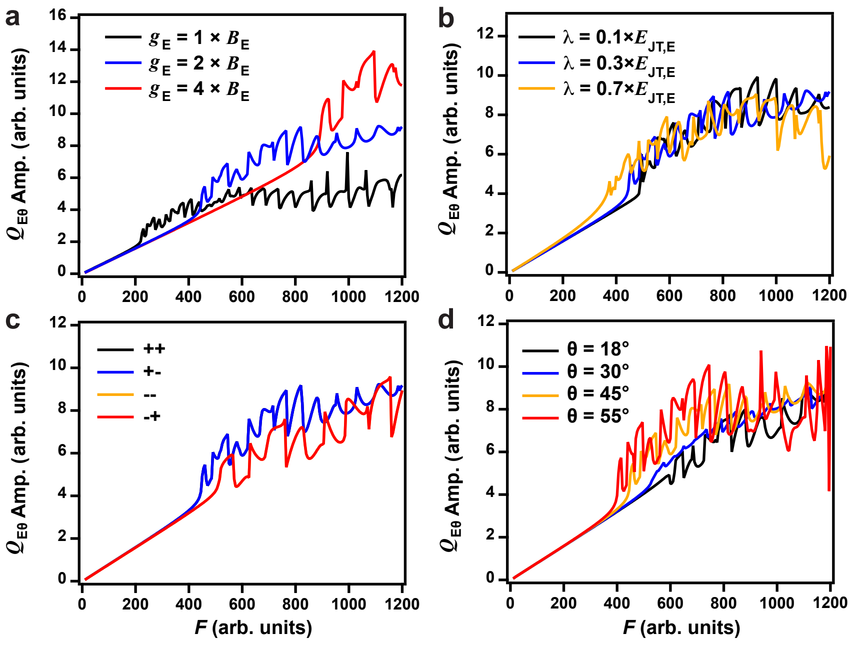

Fig. S14. Pump fluence dependence of the QOCP amplitude projected onto the $Q_{E\theta}$ coordinate as a function of **a** g_E and **b** λ. We set $B_E = \omega_0 = (2\pi) \times 4$ THz, $k_E = 0$, and $\gamma_E = 0.4$ THz. When changing g_E, λ is always set as $0.3\frac{g_E^2}{2B_E} = 0.3E_{JT,E}$; When changing λ, g_E is always set as $2B_E$. Pump fluence dependence of the QOCP amplitude projected onto the $Q_{E\theta}$ coordinate with different initial launching **c** signs and **d** directions. The first and second signs in panel **c** indicate the signs of $F(t)$ along the $Q_{E\theta}$ and $Q_{E\epsilon}$ directions, respectively. The same parameters used in panels **a** and **b** are adopted in these simulations.

Since the phonon energy B_E and lifetime γ_E are set by experiments, we examine the robustness of our model by changing the values of g_E and λ within a reasonable range as well as under different pump configurations.

We first see that modulating g_E is almost identical to rescaling the fluence-dependence curves (Fig. S14a). This is

because increasing g_E without modulating B_E and λ makes the minima in the PES deeper and farther apart without changing the shape of the local curvature in the minima. Therefore, the required fluence to overcome the barrier is proportionally higher and the required phonon amplitude is proportionally larger, while the phonon frequency exhibits little variation.

We also find that increasing λ will slightly decrease the critical fluence (Fig. S14b). This result is intuitive because increasing λ will lower the potential barrier and the amplitude of lattice distortion. Therefore, the reversal will be easier to realize. At the same time, we see a slight decrease of phonon frequency due to the λ-induced softening of the effective B_E especially around the boundary regions.

We tried to change the sign of $F(t)$ in Eq.S10. The sign of $F(t)$ along both $Q_{E\theta}$ and $Q_{E\epsilon}$ axes cooperatively determines the initial direction along which the system evolves upon impulsive excitation. $F(t)$ with the same sign along both $Q_{E\theta}$ and $Q_{E\epsilon}$ axes (i.e. both are positive or both are negative) will drive the system from the z-compressed state to the y-compressed state while $F(t)$ with opposite signs along $Q_{E\theta}$ and $Q_{E\epsilon}$ axes will drive the system to the x-compressed state. Our simulation shows that the general phonon dynamics are insensitive to the sign change (Fig. S14c).

We can also slightly change the initial launching direction of the QOCP in ($Q_{E\theta}$,$Q_{E\epsilon}$) plane by assigning different A values to the two coordinates. We can use an angle θ to parametrize the deviation from our initial assumption that $Q_{E\theta}$ and $Q_{E\epsilon}$ share the same A, i.e. $\theta = 45°$ (Fig. S14d). The fluence dependence of the QOCP amplitude is very similar despite the change of the initial launching condition. However, a change in F_c can be observed because the initial launching direction will determine the specific position where the system overcomes the barrier in the $(Q_{E\theta}, Q_{E\epsilon})$ plane.

Based on these tests, we reach a conclusion that the nonlinear fluence dependence of the QOCP amplitude that occurs upon QO switching is very robust against parameter changes in our model.

Supplementary Note 10. Possible collective lattice distortion induced by QO switching

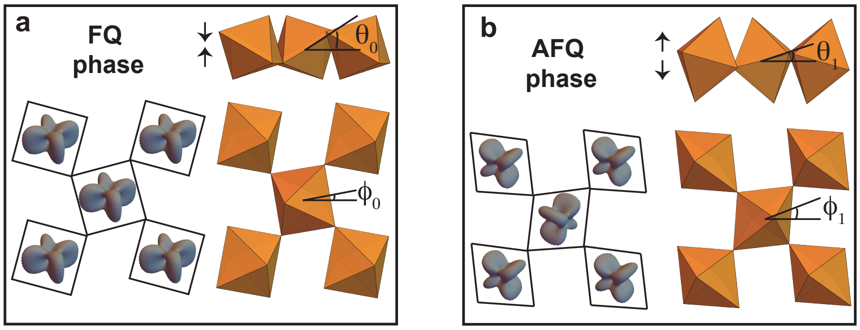

Fig. S15. Illustration of the collective pseudospin and lattice configurations of the **a,** equilibrium quadrupolar ordered phase (FQ phase) and **b,** hidden quadrupolar ordered phase (AFQ phase).

The equilibrium crystal structure of Ca_2RuO_4 is composed of an in-plane corner-shared octahedral network. The distortion of each individual octahedron upon QO switching is expected to generate a collective lattice change. For $T < T_{QO}$, X-ray spectroscopy reported a "ferroquadrupolar" (FQ) order, where all octahedra favor the d_{xy}-dominated orbital occupation and experience a tetragonal compression along z-axis (Fig. S15a) [14]. When the QO switch is induced by the driving pulse, however, each octahedron will evolve into either d_{xz}- or d_{yz}-dominated state and each octahedron will become compressed along the y- or x-axis, respectively. Due to the in-plane isotropy of each octahedron [15, 35], these two states will be reached with equal possibilities. Therefore, the octahedra may form mesoscopic domains with either y- or x-compressed octahedra. Another possible scenario is that the lattice will eventually evolve into a state with checkerboard "antiferroquadrupolar" (AFQ) order, i.e., the nearest-neighbor octahedra host opposite lattice distortions and orbital occupations (Fig. S15b). This kind of QO has been observed in manganites [36].

Previous X-ray diffraction results show that along with the Ru-O bond length change as temperature decreases, the octahedron will tilt by an angle θ along an in-plane axis bisecting the right angle between two adjacent Ru-O bonds and rotate along z-axis by an angle ϕ [15, 35]. Therefore, it is also plausible that θ and ϕ will change to accommodate the bond length change accompanied by the QO switch (right panels of Figs. S15a and b). In Note 12, our density functional theory calculations indeed reveal that the 3.7 THz QOCP involves a rotation and tilting of the octahedra [16].

Supplementary Note 11. Probe-energy-dependent datasets

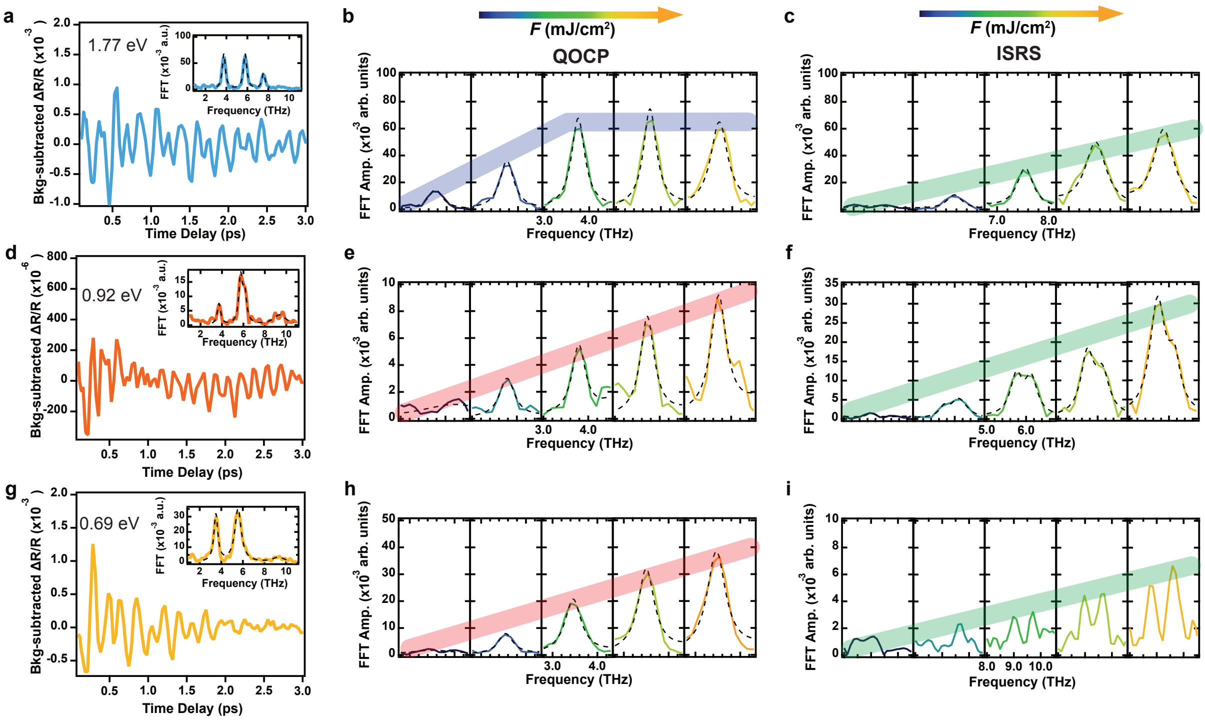

Fig. S16. **a,d,g,** Background-subtracted differential reflectivity transients at select probe energies with a pump fluence F =15 mJ/cm^2. The insets depict the FFT spectra of the corresponding transients and the dashed lines are multi-Lorentzian fits. **b,e,h,** Fluence dependence of FFT spectra of the 3.7 THz QOCP probed at 1.77 eV, 0.92 eV, and 0.69 eV. Blue and red thick lines are guides to the eye, echoing the fluence dependence of $Q_{E\epsilon}$ and $Q_{E\theta}$ modes, respectively. **c,f,i,** Fluence dependence of FFT spectra of the 7.5 probed at 1.77 eV, 5.7 and 6.1 THz probed at 0.92 eV, and 9.0 and 9.7 THz probed at 0.92 eV phonons, respectively. Green thick lines are guides to the eye, echoing the fluence dependence of ISRS phonons. Dashed lines are (multi-)Lorentzian fits.

We scanned the probe energy from 0.56 eV to 2.06 eV, through both the $d_{xy} \to d_{xz/yz}$ (α-peak) and $d_{xz/yz} \to d_{xz/yz}$ (β-peak) resonance peaks. We first comment on the probe energy dependence of different phonon amplitudes. For an electronic resonance whose energy is strongly modulated by phonons, the strongest amplitude of the phonon oscillation emerges where the first-derivative (slope) of the electronic resonance is the largest [37, 38]. By comparing the phonon traces in Fig. S2b and the optical conductivity spectra in Fig. S5, it is apparent that the phonon amplitude is the largest at the rising edge of the two peaks and vanishes when there is a peak or dip in the optical spectrum (i.e. at 1.1, 1.6, and 2.1 eV).

Since the phonons are faint at probe energies perfectly resonant with the $d_{xy} \to d_{xz/yz}$ and $d_{xz/yz} \to d_{xz/yz}$ transitions, we chose probe energies at 0.69 and 0.92 eV located at the rising edge of the α-peak, and probe energies at 1.55 and 1.77 eV located at the rising edge of the β-peak to investigate the phonon dynamics (Fig. S16). We can always unambiguously resolve the 3.7 THz QOCP regardless of the probe energies. The fluence-dependent FFT spectra of the 3.7 THz phonon at different probe energies are shown in Figs. S14b,e,h and the data in Fig. 4d are obtained from fits to these FFT spectra.

To comprehensively understand whether the phonon amplitude anomaly is unique to the 3.7 THz mode, we investigate the pump fluence dependence of every mode measured over a wide range of probe energies resonant with either the $d_{xy} \to d_{xz/yz}$ or $d_{xz/yz} \to d_{xz/yz}$ transition (Fig. S17). This comprehensive plot allows for thorough examination and cross-checking of the fluence dependence of different modes at various probe energies. The 5.7 THz mode observed at all the probe energies consistently shows a quasi-linear dependence on the pump fluence, highlighting its QO-uncoupled nature. Although the 7.5 THz mode only shows at $d_{xz/yz} \to d_{xz/yz}$-resonant probe energies, it also shows clear linear dependence on pump fluence, in contrast to the 3.7 THz mode in the same probe energy range. These observations demonstrate that both the 5.7 THz and 7.5 THz modes are not QOCPs.

The 6.1 THz, 9.0 THz, and 9.7 THz modes can only be resolved at probe energies resonant with the $d_{xy} \to d_{xz/yz}$

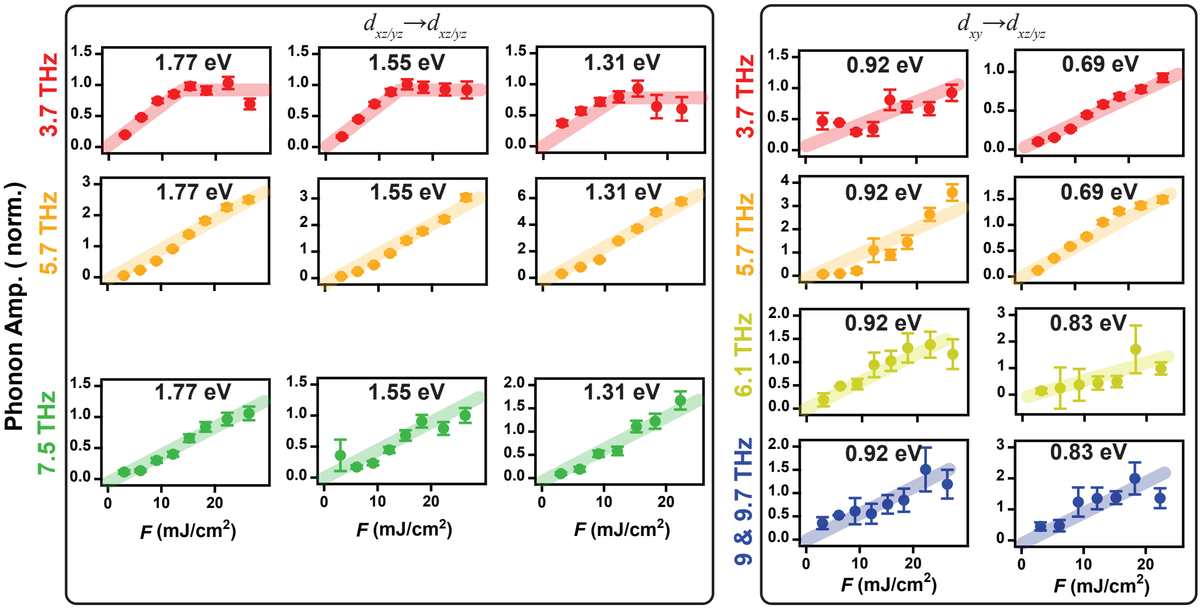

Fig. S17. Pump fluence dependence of the amplitude of various phonons probed at select energies. All the data are normalized by the maximal 3.7 THz phonon amplitude measured at the corresponding probe energies. Thick lines are guides to the eye. The amplitude of the 9 THz and 9.8 THz modes are shown together because they cannot be clearly distinguished at most probe energies. The error bars are obtained from the standard deviation of the multi-Lorentzian fitting to the FFT spectra.

transition. They all exhibit quasi-linear fluence dependence within the fitting error bars. There might be a small deviation from linearity at $F > 22$ mJ/cm^2, but the raw FFT spectra in Fig. S16 still indicate an increase with fluence without saturation. Therefore, the deviation may result from the imperfect Lorentzian fits. However, since the 3.7 THz QOCP also does not exhibit clear deviation from linearity at these probe energies, it remains possible that these modes are QOCP and they might exhibit nonlinear pump fluence dependence if they could be observed with probe energies resonant with the $d_{xz/yz} \to d_{xz/yz}$ transition.

In summary, as we claimed in the main text, the amplitudes of all the other five modes continue to scale linearly up to $F = 25$ mJ/cm^2 at all the probe energies where they can be observed. This supports our main conclusion that the 3.7 THz mode is a QOCP, while the 5.7 THz and 7.5 THz modes are not. However, we cannot definitively determine whether the 6.1 THz, 9.1 THz, and 9.7 THz modes are QOCPs, necessitating further research. Regardless, our key finding remains robust, even if more than one mode is coupled to the QO.

Supplementary Note 12. Density functional theory simulation results

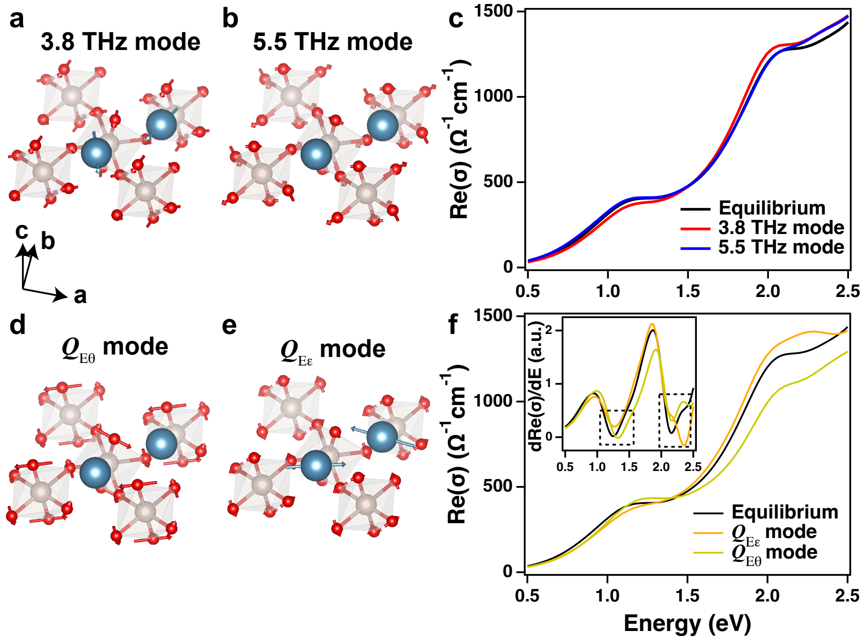

Fig. S18. **a,** DFT-calculated eigenvectors of the 3.8 THz mode and **b,** the 5.5 THz mode. Grey atoms refer to Ru. Red atoms refer to O. Blue atoms refer to Ca. The displacement vectors of each atom are denoted with arrows that are exaggerated by 10 times for clarity. **c,** Optical conductivity along the a-axis in the presence of a static lattice displacement along the eigenvectors of the aforementioned two phonons. **d,** DFT-calculated eigenvectors of the tetragonal and **e,** the orthorhombic octahedral distortions. The displacement vectors of each atom are denoted with arrows that are exaggerated by 10 times for clarity. **f,** The optical conductivity along the a-axis in the presence of a static lattice displacement along the eigenvectors of the two octahedral distortions. The first derivative of the optical spectra to show the peak position movement is shown in the inset.

We calculated the static optical conductivity spectrum using density functional theory (DFT). The real part of the optical conductivity along the a-axis exhibits two peaks at around 1 and 2 eV, matching the experimentally assigned $d_{xy}\rightarrow d_{xz/yz}$ and $d_{xz/yz}\rightarrow d_{xz/yz}$ transition peaks (Fig. S18c). We then calculated the phonon modes and found two A_{1g} phonons at 3.8 and 5.5 THz, in close agreement with the two experimentally resolved modes at 3.7 and 5.7 THz. We then simulated the real-space eigenvectors of these two modes and calculated their induced optical conductivity changes under the frozen phonon assumption (Figs. S18a-c). Assuming an oxygen atomic displacement of approximately 0.01 Å(to avoid nonlinearity) along the corresponding phonon eigenvectors, we found that the 3.8 THz mode induces a larger change in optical conductivity than the 5.5 THz mode within our probe energy range. This result demonstrates a stronger coupling between the 3.8 THz mode and the d-d transition peaks and possibly explains the direct coupling of the 3.8 THz mode to the QO.

We also calculated the optical conductivity change induced by the two kinds of octahedral eigen-distortions, namely the tetragonal distortion $Q_{E\theta}$ and the orthorhombic distortion $Q_{E\epsilon}$ (Figs. S18d-f). We find both modes with similar amplitudes of oxygen atomic displacement can generate prominent changes in the two optical transition peaks. To examine the peak position change induced by the two kinds of distortions more definitively, we calculated the first derivative of the optical conductivity over the probe energy range. The two dips enclosed by the dashed rectangles in the inset of Fig. S15f represent the position of the two transition peaks. Our results show that the $Q_{E\theta}$ mode displaces the lower energy transition peak more than the $Q_{E\epsilon}$ mode, whereas the latter shifts the position of the higher energy peak more. These observations match our expectation as discussed in the main text. Therefore, our DFT results support our conjecture that scanning the probe energy from the $d_{xy}\rightarrow d_{xz/yz}$ to $d_{xz/yz}\rightarrow d_{xz/yz}$ transition peak enables a selective coupling to the $Q_{E\theta}$ and $Q_{E\epsilon}$ component of the phonon, respectively.

Supplementary Note 13. Relationship between the A_g coherent phonons and octahedral eigenmodes Q's

The QO transition is driven by the 3.7 THz phonon mode, which can be mapped onto the two octahedral eigen-deformations $Q_{E\theta}$ and $Q_{E\epsilon}$. This mapping theoretically applies to all phonon eigenmodes. Considering a perfect octahedron with O_h point group symmetry, any inversion-symmetric distortion can be represented as a superposition of eigen-distortions respecting A_{1g}, A_{2g}, E_g, T_{1g} or t_{2g} symmetries. Therefore, these eigenmodes form a complete orthonormal basis for any type of distortion of an isolated octahedron. For the t_{2g} manifold electrons, only six different distortions respecting A_{1g}, E_g ($Q_{E\theta}$, $Q_{E\epsilon}$), and t_{2g} ($Q_{T\eta}$, $Q_{T\xi}$, $Q_{T\zeta}$) symmetries are allowed to couple to the electrons, whose spatial configurations are displayed in Fig. S9.

In other words, any t_{2g}-electron-coupled phonon eigenmodes, regardless of their symmetries with respect to the entire lattice, is a superposition of the six octahedral eigenfunctions: $Q = a_A Q_A + a_\theta Q_{E\theta} + a_\epsilon Q_{E\epsilon} + a_\eta Q_{T\eta} + a_\xi Q_{T\xi} + a_\zeta Q_{T\zeta}$, where the $a$âs represent the ratio of different components. Since Q_A is fully symmetric and JT inactive, the leading-order bilinear JT coupling term can be expressed as: $g_E(a_\theta Q_{E\theta}\tau_{E\theta} + a_\epsilon Q_{E\epsilon}\tau_{E\epsilon}) + g_T(a_\eta Q_{T\eta}\tau_{T\zeta} + a_\xi Q_{T\xi}\tau_{T\zeta} + a_\zeta Q_{T\zeta}\tau_{T\zeta})$, where $g$âs are the JT coupling constants and τâs are the electronic QO parameters. In transition metal oxides with only static E_g (tetragonal, orthorhombic) distortions like Ca_2RuO_4, the t_{2g} terms can be omitted because g_T is significantly smaller than g_E [30, 31]. Consequently, the coupling strength between the QO and any phonon mode is determined by $g(a_\theta Q_\theta \tau_\theta + a_\epsilon Q_\epsilon \tau_\epsilon)$ (subscript âEâ is dropped for simplicity).

Having established the theoretical foundation, immediate questions are how the 3.7 THz QOCP is mapped to Q_θ and Q_ϵ and why the other modes are not QOCP. To address these questions, we utilize two methods to demonstrate how the 3.7 THz mode can be mapped onto Q_θ and Q_ϵ, i.e. obtain a_θ and a_ϵ. Furthermore, we apply the same analysis to the 5.7 THz phonon as a comparison, which is an unambiguously QO-uncoupled mode due to its consistent linear fluence dependence across the entire probe energy range.

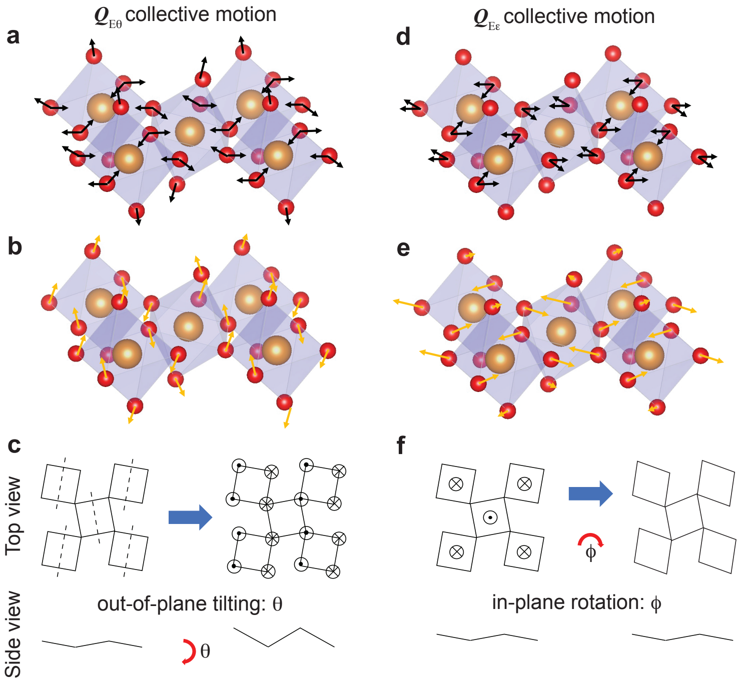

Fig. S19. **a,** Schematic of the tetragonal distortion in the lattice. Only one layer of Ru-O octahedra is shown for clarity. Black arrows mark the desired displacement of the oxygen atoms, which are shared by two octahedra, in each individual octahedron. **b,** Schematic of the eigenvector of the tetragonal distortion in the lattice as marked by the yellow arrows, which are the sum of black arrows at each atomic position. **c,** Schematics showing the out-of-plane tilting of octahedra induced by the tetragonal distortion from the top and side views. Dashed lines indicate the tilting axes. **d-f,** Same as **a-c** but for the orthorhombic distortion.

First, we qualitatively analyzed the components of Q_θ and Q_ϵ, i.e. a_θ and a_ϵ, by examining the amplitude and direction of the phonon eigenvectors. Ca_2RuO_4 is composed of in-plane corner-shared octahedra. As the octahedra undergo distortion, a collective tilting or rotation must follow to accommodate the bond length changes. By considering

these geometric constraints, we imposed the required length change in the Ru-O bond of Q_θ and Q_ϵ and simulated the eigenvectors of Q_θ and Q_ϵ within the entire lattice after accommodating the bond length changes. Interestingly, we find that the eigenvector of the tetragonal distortion Q_θ mainly exhibits an out-of-plane component, indicating a collective tilting of the octahedra along an in-plane axis (Figs. S19a-c). On the other hand, the eigenvector of the orthorhombic distortion Q_ϵ mostly shows an in-plane component, suggesting a collective rotation of all the octahedra along an out-of-plane axis through the center Ru atoms (Figs. S19d-f). As a result, analyzing the direction of the eigenvectors allows us to roughly distinguish the components of Q_θ and Q_ϵ for different phonons. Another distinguishing feature between the two modes is the amplitude of displacement of the apical oxygens. The length of the apical Ru-O bonds remains unchanged in the Q_ϵ mode, so the apical oxygen atoms only displace slightly following the rotation of the octahedron. However, the net displacement of the apical oxygen of the Q_θ mode is significantly larger, due to the combination of tilting of the octahedron and the required length change for Q_θ.

We now examine the eigenvectors of the 3.7 THz and 5.7 THz modes obtained from our DFT simulations (Figs. S18a,b) using the aforementioned two criteria. We find that the eigenvectors of the 3.7 THz mode displays a notably larger in-plane displacement of the planar oxygens and a larger displacement of the apical oxygens compared to the 5.7 THz mode. These two observations suggest a larger component of both Q_θ and Q_ϵ components in the 3.7 THz mode compared to the QO-uncoupled 5.7 THz mode. Given the larger projection on Q_θ and Q_ϵ, the 3.7 THz mode appears to be a more suitable candidate for QOCP than the 5.7 THz mode.

To more quantitatively obtain a_θ and a_ϵ, we calculated the length change in the four planar Ru-O bonds and two apical Ru-O bonds based on the DFT results. Theoretically, Q_θ induces a length change with the same amplitude (d_θ) and sign in the four planar Ru-O bonds, while Q_ϵ results in a length change with the same amplitude (d_ϵ) and opposite signs in the neighboring planar Ru-O bonds. Consequently, the net length change of two neighboring planar Ru-O bonds should be $d_\theta + d_\epsilon$ and $d_\theta - d_\epsilon$ when both distortions are present. The apical Ru-O bond change, on the other hand, predominantly originates from Q_θ. Since $d_\theta : d_\epsilon = a_\theta : a_\epsilon$, analyzing the length change of different Ru-O bonds provides a direct quantitative way to determine a_θ and a_ϵ. With identical Ca atom displacements for both modes, we find that the neighboring planar Ru-O bond length change ratio is roughly $1 : 2.36$ and $1.29 : 1.32$ for the 3.7 THz and 5.7 THz modes, respectively. This indicates that $a_\theta : a_\epsilon = 1.68 : 0.68$ and $1.31 : 0.01$ for the two modes, respectively. In other words, the projection on Q_θ (a_θ) of the 3.7 THz mode is nearly $1.68/1.31 = 1.3$ times higher than that of the 5.7 THz mode. This is further corroborated by the length change in the apical Ru-O bond, with the 3.7 THz mode exhibiting nearly double the bond length change compared to the 5.7 THz mode. On the other hand, the projection on Q_ϵ (a_ϵ) of the 5.7 THz mode is nearly zero. The negligible composition of Q_ϵ in the 5.7 THz mode explains the absence of phonon amplitude saturation (which is most apparent in Q_ϵ) in the 5.7 THz mode.

Based on the aforementioned qualitative and quantitative analysis, we have demonstrated that both a_θ and a_ϵ are higher in the 3.7 THz mode. However, the formula $g(a_\theta Q_\theta \tau_\theta + a_\epsilon Q_\epsilon \tau_\epsilon)$ indicates that the coupling strength to the QO is also determined by g in addition to a_θ and a_ϵ. Interestingly, our DFT simulation (Fig. S18c) has shown that the 3.7 THz mode induces a larger change in optical conductivity than the 5.5 THz mode at the probe energies sensitive to both Q_θ and Q_ϵ modes. This result indicates a larger g of the 3.7 THz mode, further supporting that the 3.7 THz mode is strongly coupled to the QO.

Again, we note that Q_θ and Q_ϵ are not actual phonon eigenmodes of Ca_2RuO_4 but two eigen-deformations of an octahedron. For the QOCP, which can map onto Q_θ and Q_ϵ, they both have the same frequency at 3.7 THz. Similarly, for the 5.7 THz QO-uncoupled mode, which can also map onto Q_θ and Q_ϵ but with smaller components, these two modes will also have the same frequency at 5.7 THz. Therefore, they will always have the same frequency as the phonon mode they derive from.

Supplementary Note 14. Justification of the impulsive excitation of QOCP

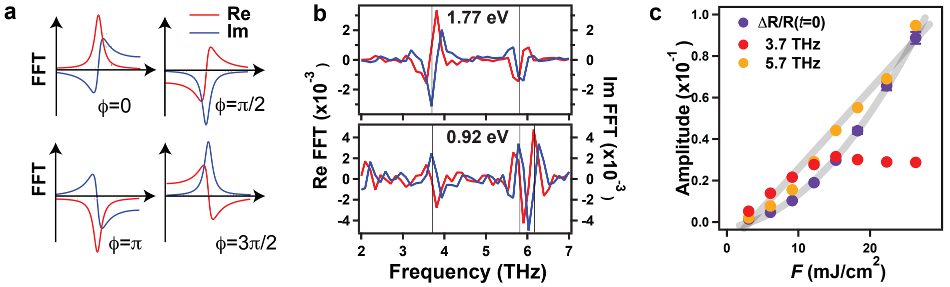

Fig. S20. **a,** Schematics of real and imaginary FFT spectra of damped cosinusoidal functions of different phases as denoted. **b,** Complex FFT spectra measured with a pump fluence of 9 mJ/cm^2 and 1.77 eV and 0.92 eV probes. Thin lines indicate the phonon positions. **c,** Fluence dependence of the amplitude of $\Delta R/R(t=0)$, the 3.7 THz phonon, and the 5.7 THz phonon measured at 1.77 eV probe. Gray lines are linear and quadratic fits. The error bars are obtained from the standard deviation of fitting.

Impulsive stimulated Raman scattering (ISRS) occurs in the transparent regime, exciting coherent phonons with a sinusoidal oscillation [23]. On the other hand, displacive excitation of coherent phonons (DECP) relies on real electronic excitations, which modifies the PES and dominates the absorbing regime, resulting in a cosinusoidal oscillation of the phonons [32]. To ensure predominantly impulsive excitation, we choose a pump photon energy well below the Mott gap and far away from phonon resonances to maximize (minimize) transmission (absorption coefficient). Our selection is supported by previous optical spectroscopy measurements, demonstrating at least one order of magnitude higher transmission and nearly 16 times smaller absorption coefficient at the pump energy (0.3 eV), as compared to 1.1 eV where the first absorption peak occurs [26, 39].

The assumption that the QOCP is impulsively excited is further justified by two observations. First, the phase of the 3.7 THz mode is close to $\pm\pi/2$. We directly complex Fourier transform the background-subtracted transient reflectivity traces at different probe energies. Assuming a damped cosinusoidal function $A\exp(-t/\tau)\cos(\omega t+\phi)$, where A, τ, ω, and ϕ are the amplitude, lifetime, frequency, and phase of the phonon, the phase ϕ can be directly obtained by simultaneously comparing the real and imaginary part of the FFT spectra as shown by the schematics in Fig. S20a. Our results show that the FFT spectra of QOCP better match the spectra of $\phi = \pm\pi/2$ at probe energies resonant with both α-peak (0.92 eV) and β-peak (1.77 eV), corroborating the ISRS mechanism (Fig. S20b).

Second, the linear pump fluence dependence of QOCP amplitude before saturation argues against the prediction of the DECP mechanism but matches the prediction of the ISRS mechanism. The DECP mechanism predicts a phonon amplitude proportional to carrier density n. In the above-gap pumping case, carriers are generated through linear absorption and n is proportional to F; in the subgap pumping case, carriers are nonlinearly generated through either multi-photon absorption or quantum tunneling processes, which gives rise to a superlinear F-dependence of n. Previous work on Ca_2RuO_4 already showed that subgap pumping leads to nonlinear carrier generation [17], manifested through a superlinear fluence dependence of $\Delta R/R(t=0)$, which is proportional to n (Fig. S20c). Therefore, if the QOCP is launched displacively, its amplitude should scale superlinearly with F like $\Delta R/R(t=0)$. However, we observed a linear fluence dependence of the QOCP before its amplitude saturates, thus refuting the DECP scenario. On the other hand, the impulsively excited phonon is stimulated through a two-photon Raman scattering process, whose amplitude is proportional to the square of the electric field strength E^2 (i.e. F), independent of the pump photon energy. This is consistent with our observation. Consequently, the evidence once again supports the dominance of the ISRS mechanism over DECP.

Nevertheless, it is important to acknowledge that despite the QOCP being predominantly impulsively excited, we cannot completely exclude a small finite contribution of DECP. Importantly, however, even if the QOCP is displacively excited, the switch of hidden QO would still occur and our main conclusions would remain unchanged. This is because the QO switch is induced by the coherent phonon oscillation and is independent of the specific mechanism that drives the phonon at $t=0$.

Supplementary Note 15. Temperature, probe energy, and fluence dependence of the QOCP phase

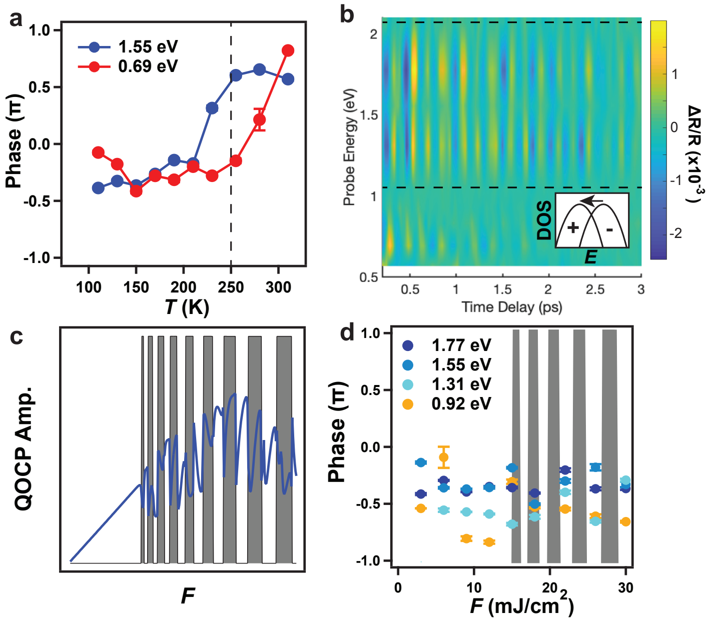

Fig. S21. **a,** Temperature dependence of the 3.7 THz phonon phase measured with a pump fluence of 15 mJ/cm^2 and probe at 1.55 eV and 0.69 eV. The dashed line marks T_{QO}. **b,** Probe energy dependent background-subtracted reflectivity transient spectrum measured with a pump fluence of 12 mJ/cm^2 at 80 K. Dashed lines denote the positions of α- and β-peaks. Inset shows if phonon modulates the position of the electronic resonance, the measured phase below and above the peak will be opposite. **c,** Fluence dependence of the QOCP amplitude from the simulation. Gray shaded area marks the fluence range where the final metastable state is a different QO, while white shaded area marks the fluence range where the final metastable state is the initial QO. **d,** Fluence dependence of the 3.7 THz phonon phase measured at select probe energies at 80 K. The error bars are obtained from the standard deviation of the multi-Lorentzian fitting to the FFT spectra.

First, we discuss the temperature dependence of the QOCP phase. As reported in Ref.[16], the π-phase flip stems from a static antipolar distortion of the apical oxygen atoms developing around T_{QO}. Therefore, this phase flip should also manifest in our experiment. As expected, our temperature-dependent 0.3 eV pump experiment shows a similar π-phase flip around T_{QO} with probe energies resonant with either α- or β-peaks (Fig. S21a), in agreement with Ref.[16].

We next comment on the probe energy dependence of the phonon phase. For an electronic resonance whose energy is strongly modulated by phonons, the phonon oscillation reaches its peak amplitude where the first-derivative (slope) of the electronic resonance is the largest [37, 38]. Moreover, the phonon oscillation should host opposite phases below and above the peak position, due to the phonon modulation of the position of the electronic resonance (Fig. S21b inset). By comparing the phonon time traces (Fig. S21b) and the optical conductivity spectra (Fig. S5a), it is apparent that not only the phonon amplitude is large when the probe energy coincides with the rising edge of the two peaks and vanishes when there is a peak or dip in the optical spectrum (i.e. at 1.1, 1.6, and 2.1 eV), but a π-phase difference between the phonon phase below and above the α-peak position can also be resolved. This phenomenon, signifying the position of electronic resonances, has been observed elsewhere [38].

Moving on to the fluence dependence of the QOCP, we need to first emphasize that the phonon phase is ill-defined for an anharmonic potential, especially in the vicinity of a photo-induced phase transition as in our case. There are two factors preventing us from drawing a definitive conclusion from the experimental results. First, according to the simulation, the phonon phase should deviate from $\pi/2$ when the QO switch occurs. However, due to the dynamical nature of the transition, the phase shift only occurs after the switch is completed, taking at least half of the phonon period. Moreover, the initial direction of the phonon oscillation, unlike the cases of changing temperature or probe energy, remains unchanged as the initial static condition is independent of pump fluence. In other words, the phonon phase evolves as a function of time, making a strict definition of phonon phase challenging. Second, as discussed in Note 9, the system may transiently traverse multiple PES minima when the pump fluence is high. Moreover, as

shown by a more comprehensive dynamical simulation using our microscopic model, the final metastable state, which is either the initial or a different QO, sensitively depends on the pump fluence (Fig. S21c white and gray regions). Therefore, in the gray fluence range where the final state is a different QO, the phonon phase will deviate from the low fluence regime where no transition occurs. However, in the white regions where the transition to a different order is only transient and the system finally relaxes back into the original QO, the long-time phonon phase should still be nearly identical to that of the low fluence regime. This complexity precludes obtaining a meaningful phonon phase. Due to these difficulties and our limited fluence sampling, we refrain from drawing any quantitative conclusions from our experimental data (Fig. S21d).

Supplementary Note 16. Estimate of the light-induced atomic displacement

To estimate the atomic displacement induced by the impulsive stimulus, we use the following expression [23, 40, 41]:

$$U_{A_1} \approx \sqrt{\frac{3.8 \times 10^{-3} B_{A_1} F}{\rho \nu_{A_1} D|\varepsilon|} \frac{\partial \varepsilon_1}{\partial \omega}} \tag{S11}$$

where $\varepsilon = \varepsilon_1 + i\varepsilon_2$ is the complex dielectric constant ($|\varepsilon| \sim 7$), ν_{A_1} is the phonon frequency (3.7 THz), $\frac{\partial \varepsilon_1}{\partial \omega}$ (~ 5 eV^{-1}), $D = (\frac{1}{R})\frac{\partial R}{\partial E}$ (~ 0.1 eV^{-1}) [26, 39], and ρ (2.69 amu/Å^3) is the density of mass, B_{A_1} is the phonon amplitude in transient reflectivity ($\sim 3 \times 10^{-3}$), and F is the fluence of the pump pulse (~ 15 mJ/cm^2). This gives rise to an atomic displacement of ~ 0.02 Å, in qualitative agreement with the length difference between the planar and apical Ru-O bonds ~ 0.03 Å[15, 35] — according to our theory, as the quadrupolar order is switched from d_{xy}-dominated to $d_{xz/yz}$-dominated, the bond length between the planar and the apical Ru-O bonds should also undergo a switch. The qualitative agreement between the estimated value and the real bond difference serves as evidence that the transient QO transition is indeed achievable at the critical pump fluence employed in our study.

Supplementary Note 17. Robustness of the QO switch against nonlinear doublon-holon generation

To impulsively excite Raman active phonons with large amplitude while minimizing heating due to absorption, we choose the pump photon energy well below the Mott gap and far away from phonon resonances. However, previous work studying the electronic responses of the same material under identical conditions reveals that despite the subgap pumping, doublon-holon pairs are still generated nonlinearly [17]. Carrier excitation may transiently modulate the PES, leading to a displacive excitation of phonons. However, the QOCP is predominantly excited by impulsive excitation based on two observations, which is justified in Note 14. One observation is that the phase of the QOCP is close to $\pm\pi/2$, matching the expectation for an impulsively excited phonon and arguing against the displacive excitation scenario. The second observation is that the QOCP amplitude scales linearly with fluence before it saturates, which is inconsistent with a displacive excitation mechanism. This is because the amplitude of phonons excited displacively scales linearly with carrier density, but the carrier density exhibits a superlinear fluence dependence for subgap pumping either through multi-photon or quantum tunneling processes. Rather, our results are consistent with the impulsive Raman scattering process (Fig. S20). Therefore, the nonlinear carrier generation does not affect the predominantly impulsive excitation of QOCP and the QO switch. Moreover, we would like to note that even if the excitation were displacive, the QO switch can still be induced by the coupled phonon excitation and our main conclusion is still valid. This is because the QO switch is induced by the coherent phonon oscillation and is independent of the specific mechanism that drives the phonon at $t = 0$. Therefore, the nonlinear carrier generation does not affect our main conclusion that a coherent phonon can induce a switch to a hidden quadrupolar-ordered state.

* These authors contributed equally to this work
† Present address: Institut für Physik und IRIS Adlershof, Humboldt-Universität zu Berlin, Zum Großen Windkanal, Berlin, Germany
‡ email: dhsieh@caltech.edu

[1] Stamokostas, G. L. & Fiete, G. A. Mixing of $t_{2g} - e_g$ orbitals in 4d and 5d transition metal oxides. *Phys. Rev. B* **97**, 085150 (2018).
[2] Georges, A., Medici, L. d. & Mravlje, J. Strong correlations from Hund's coupling. *Annu. Rev. Condens. Matter Phys.* **4**, 137–178 (2013).
[3] Khaliullin, G. Excitonic magnetism in van vleck–type d^4 Mott insulators. *Phys. Rev. Lett.* **111**, 197201 (2013).
[4] Jain, A. *et al.* Higgs mode and its decay in a two-dimensional antiferromagnet. *Nat. Phys.* **13**, 633–637 (2017).
[5] Das, L. *et al.* Spin-orbital excitations in Ca_2RuO_4 revealed by resonant inelastic x-ray scattering. *Phys. Rev. X* **8**, 011048 (2018).
[6] Gretarsson, H. *et al.* Observation of spin-orbit excitations and Hund's multiplets in Ca_2RuO_4. *Phys. Rev. B* **100**, 045123 (2019).
[7] Souliou, S.-M. *et al.* Raman scattering from Higgs mode oscillations in the two-dimensional antiferromagnet Ca_2RuO_4. *Phys. Rev. Lett.* **119**, 067201 (2017).
[8] Mizokawa, T. *et al.* Spin-orbit coupling in the Mott insulator Ca_2RuO_4. *Phys. Rev. Lett.* **87**, 077202 (2001).
[9] Sutter, D. *et al.* Hallmarks of Hund's coupling in the Mott insulator Ca_2RuO_4. *Nat. Commun.* **8**, 15176 (2017).
[10] Streltsov, S. V. & Khomskii, D. I. Jahn-Teller effect and spin-orbit coupling: Friends or foes? *Phys. Rev. X* **10**, 031043 (2020).
[11] Liu, H. & Khaliullin, G. Pseudo-Jahn-Teller effect and magnetoelastic coupling in spin-orbit Mott insulators. *Phys. Rev. Lett.* **122**, 057203 (2019).
[12] Chen, G., Pereira, R. & Balents, L. Exotic phases induced by strong spin-orbit coupling in ordered double perovskites. *Phys. Rev. B* **82**, 174440 (2010).
[13] Chen, G. & Balents, L. Spin-orbit coupling in d^2 ordered double perovskites. *Phys. Rev. B* **84**, 094420 (2011).
[14] Zegkinoglou, I. *et al.* Orbital ordering transition in Ca_2RuO_4 observed with resonant x-ray diffraction. *Phys. Rev. Lett.* **95**, 136401 (2005).
[15] Friedt, O. *et al.* Structural and magnetic aspects of the metal-insulator transition in $Ca_{2-x}Sr_xRuO_4$. *Phys. Rev. B* **63**, 174432 (2001).
[16] Lee, M.-C. *et al.* Abnormal phase flip in the coherent phonon oscillations of Ca_2RuO_4. *Phys. Rev. B* **98**, 161115 (2018).
[17] Li, X. *et al.* Keldysh space control of charge dynamics in a strongly driven Mott insulator. *Phys. Rev. Lett.* **128**, 187402 (2022).
[18] Rho, H., Cooper, S. L., Nakatsuji, S., Fukazawa, H. & Maeno, Y. Lattice dynamics and the electron-phonon interaction in Ca_2RuO_4. *Phys. Rev. B* **71**, 245121 (2005).
[19] Hunsche, S., Wienecke, K., Dekorsy, T. & Kurz, H. Impulsive softening of coherent phonons in tellurium. *Phys. Rev. Lett.* **75**, 1815–1818 (1995).
[20] Tangney, P. & Fahy, S. Calculations of the A_1 phonon frequency in photoexcited tellurium. *Phys. Rev. Lett.* **82**, 4340–4343 (1999).
[21] Hase, M., Kitajima, M., Nakashima, S.-i. & Mizoguchi, K. Dynamics of coherent anharmonic phonons in bismuth using high density photoexcitation. *Phys. Rev. Lett.* **88**, 067401 (2002).
[22] Gorelov, E. *et al.* Nature of the Mott transition in Ca_2RuO_4. *Phys. Rev. Lett.* **104**, 226401 (2010).
[23] Stevens, T. E., Kuhl, J. & Merlin, R. Coherent phonon generation and the two stimulated Raman tensors. *Phys. Rev. B* **65**, 144304 (2002).
[24] Misochko, O. V. & Lebedev, M. V. Coherent lattice dynamics in opaque crystals: Testing the adequacy of two-tensor model. *Phys. Rev. B* **94**, 184307 (2016).
[25] Harter, J. W. *et al.* Evidence of an improper displacive phase transition in $Cd_2Re_2O_7$ via time-resolved coherent phonon spectroscopy. *Phys. Rev. Lett.* **120**, 047601 (2018).
[26] Jung, J. H. Raman scattering and optical absorption studies of an orbital ordered Ca_2RuO_4. *Solid State Commun.* **133**, 103–107 (2005).
[27] Qi, T. F., Korneta, O. B., Parkin, S., Hu, J. & Cao, G. Magnetic and orbital orders coupled to negative thermal expansion in Mott insulators $Ca_2Ru_{1-x}M_xO_4$ (M = Mn and Fe). *Phys. Rev. B* **85**, 165143 (2012).
[28] Kawasaki, S. *et al.* Thermal diffusivity of the Mott insulator Ca_2RuO_4 in a non-equilibrium steady state. *J. Phys. Soc. Japan* **90**, 063601 (2021).
[29] Bersuker, I. The Jahn-Teller Effect. Cambridge University Press, 2006.
[30] Iwahara, N., Vieru, V. & Chibotaru, L. F. Spin-orbital-lattice entangled states in cubic d^1 double perovskites. *Phys. Rev. B* **98**, 075138 (2018).
[31] Plotnikova, E. M., Daghofer, M., van den Brink, J. & Wohlfeld, K. Jahn-Teller effect in systems with strong on-site spin-orbit coupling. *Phys. Rev. Lett.* **116**, 106401 (2016).
[32] Zeiger, H. J. *et al.* Theory for displacive excitation of coherent phonons. *Phys. Rev. B* **45**, 768–778 (1992).
[33] Kuznetsov, A. V. & Stanton, C. J. Theory of coherent phonon oscillations in semiconductors. *Phys. Rev. Lett.* **73**, 3243–3246 (1994).

[34] Melnikov, A., Misochko, O. & Chekalin, S. Generation of coherent phonons in bismuth by ultrashort laser pulses in the visible and NIR: Displacive versus impulsive excitation mechanism. *Phys. Lett. A* **375**, 2017 – 2022 (2011).

[35] Braden, M., André, G., Nakatsuji, S. & Maeno, Y. Crystal and magnetic structure of Ca_2RuO_4 magnetoelastic coupling and the metal-insulator transition. *Phys. Rev. B* **58**, 847–861 (1998).

[36] Tokura, Y. & Nagaosa, N. Orbital physics in transition-metal oxides. *Science* **288**, 462–468 (2000).

[37] Ergeçen, E. *et al.* Magnetically brightened dark electron-phonon bound states in a van der Waals antiferromagnet. *Nat. Commun.* **13**, 98 (2022).

[38] Bae, Y. J. *et al.* Exciton-coupled coherent magnons in a 2D semiconductor. *Nature* **609**, 282–286 (2022).

[39] Jung, J. H. *et al.* Change of electronic structure in Ca_2RuO_4 induced by orbital ordering. *Phys. Rev. Lett.* **91**, 056403 (2003).

[40] DeCamp, M. F., Reis, D. A., Bucksbaum, P. H. & Merlin, R. Dynamics and coherent control of high-amplitude optical phonons in bismuth. *Phys. Rev. B* **64**, 092301 (2001).

[41] Kamaraju, N., Kumar, S., Anija, M. & Sood, A. K. Large-amplitude chirped coherent phonons in tellurium mediated by ultrafast photoexcited carrier diffusion. *Phys. Rev. B* **82**, 195202 (2010).
